# Anti‐HER2 Super Stealth Immunoliposomes for Targeted‐Chemotherapy

**DOI:** 10.1002/adhm.202301650

**Published:** 2023-08-30

**Authors:** Elena Canato, Antonella Grigoletto, Ilaria Zanotto, Tommaso Tedeschini, Benedetta Campara, Giovanna Quaglio, Giuseppe Toffoli, Delia Mandracchia, Alberto Dinarello, Natascia Tiso, Francesco Argenton, Katia Sayaf, Maria Guido, Daniela Gabbia, Sara De Martin, Gianfranco Pasut

**Affiliations:** ^1^ Department Pharmaceutical and Pharmacological Sciences University of Padova Via Marzolo 5 Padova 35131 Italy; ^2^ Experimental and Clinical Pharmacology Centro di Riferimento Oncologico di Aviano (CRO) IRCCS Via Franco Gallini n. 2 Aviano 33081 Italy; ^3^ Department of Molecular and Translational Medicine University of Brescia Brescia 25123 Italy; ^4^ Department of Biology University of Padova Via U. Bassi 58/B Padova 35131 Italy; ^5^ Department Surgery, Oncology and Gastroenterology University of Padova Via Giustiniani 2 Padova 35131 Italy; ^6^ Department of Medicine‐DIMED University of Padova Padua 35128 Italy; ^7^ Department of Pathology Azienda ULSS2 Marca Trevigiana Treviso 31100 Italy

**Keywords:** anticancer therapy, doxorubicin, drug targeting, liposomes, targeted chemotherapy, trastuzumab

## Abstract

Liposomes play an important role in the field of drug delivery by virtue of their biocompatibility and versatility as carriers. Stealth liposomes, obtained by surface decoration with hydrophilic polyethylene glycol (PEG) molecules, represent an important turning point in liposome technology, leading to significant improvements in the pharmacokinetic profile compared to naked liposomes. Nevertheless, the generation of effective targeted liposomes—a central issue for cancer therapy—has faced several difficulties and clinical phase failures. Active targeting remains a challenge for liposomes. In this direction, a new Super Stealth Immunoliposomes (SSIL2) composed of a PEG‐bi‐phospholipids derivative is designed that stabilizes the polymer shielding over the liposomes. Furthermore, its counterpart, conjugated to the fragment antigen‐binding of trastuzumab (Fab’_TRZ_‐PEG‐bi‐phospholipids), is firmly anchored on the liposomes surface and correctly orients outward the targeting moiety. Throughout this study, the performances of SSIL2 are evaluated and compared to classic stealth liposomes and stealth immunoliposomes in vitro in a panel of cell lines and in vivo studies in zebrafish larvae and rodent models. Overall, SSIL2 shows superior in vitro and in vivo outcomes, both in terms of safety and anticancer efficacy, thus representing a step forward in targeted cancer therapy, and valuable for future development.

## Graphical abstract

Stable interaction of targeting agents over the surface of super stealth liposomes, delivering an anticancer drug, improves the cancer treatment outcomes and reduces side‐effects.

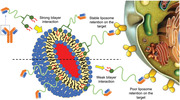

## 1 Introduction

Anticancer‐targeted chemotherapy is an intense field of study that holds the promise of selective and effective treatment of tumors. Drug delivery scientists have investigated a vast number of approaches for achieving the aim of selective tumor delivery, but unfortunately, although the promising preclinical results, none of the proposed ligand‐targeted (or active targeting) delivery systems reached the clinical practice.^[^ ^1^, ^2^ ^]^ So far, only non‐targeted nanomedicines have been approved for human use. In these cases, the advantages are mainly related to a reduction of toxic side effects due to the free drugs. Antibody‐drug conjugates (ADCs) are the only exception that achieved the goal of selective tumor therapy with very good results. They represent a peculiar class of nanomedicines in which a limited number of drug molecules are conjugated per antibody unit and whose approval is based on the biological license applications (BLA) process.^[^ ^3^ ^]^

Liposomes have been the first nanomedicine approved for clinical use and are still considered a useful and versatile approach that can be tailored to specific needs by tuning their size, morphology, composition, surface modification, etc.^[^ ^4^, ^5^ ^]^ To obtain a selective delivery to solid cancers, the approved liposomes loaded with cytotoxic drugs can rely only on passive tumor accumulation by exploiting the enhanced vascular permeability and retention (EPR) effect, due to their size.^[^ ^6^ ^]^ However, the real benefits of EPR effects for significant tumor selectivity are still under debate^[^ ^7^, ^8^, ^9^ ^]^ and some evidence suggest that only a minor fraction of the injected dose of a nanomedicine can reach the cancer site by exploiting the EPR effect.^[^ ^1^ ^]^ Although surface decoration with hydrophilic polymers, commonly PEG, confers “stealth” properties, thereby reducing the clearance rate and increasing the circulation half‐life of the nanocarrier,^[^ ^10^ ^]^ such improvement is not achieving the goal of a selective tumor targeting.

Liposome surface functionalization with antibodies might enhance the accumulation of the nanomedicine into tumors through antibody‐mediated recognition of specific antigens overexpressed on the membrane of cancer cells. However, the coupling of the whole antibody resulted to be highly immunogenic, with a reduction of the mean residence time of the immunoliposomes in the bloodstream,^[^ ^11^ ^]^ attributable to several mechanisms such as Fc‐mediated phagocytosis, opsonization, complement‐mediated cytotoxicity, antibody‐dependent cellular cytotoxicity (ADCC). Advancement in monoclonal antibody engineering allowed the use of mAb fragments able to preserve the antigen binding characteristic, like Fab’ or the single chain fragment variable (scFv) fragments.^[^ ^12^, ^13^ ^]^

**Figure 1:**
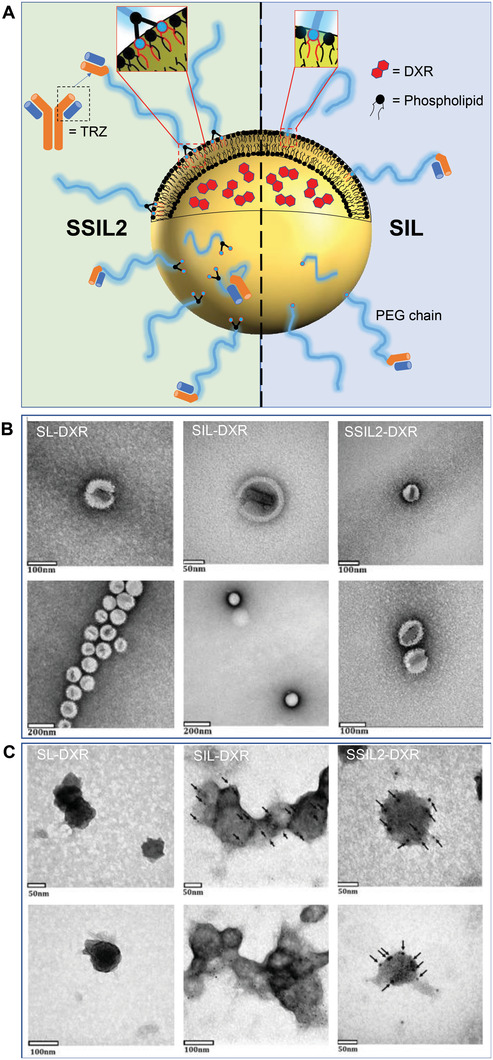
Schematic representation and TEM images of liposomes. A) Drawing of SSIL2 and SIL highlighting the different anchoring of PEG chains on the phospholipid bilayer. B) TEM characterization of SL‐DXR, SIL‐DXR, and SSIL2‐DXR. C) Immunolabeling of targeted liposomes (SIL and SSIL2) compared to non‐targeted liposomes (SL) as the negative control. The black dots indicated by the arrows correspond to the gold nanoparticles (5 nm) conjugated to a secondary anti‐mouse antibody binding the primary anti‐Trastuzumab antibody, thus allowing indirect visualization of the Fab’ fragments on the surface of liposomes. The gold nanoparticles were not detected in non‐targeted liposomes.

Here, the Fab’ derived from Trastuzumab (TRZ), a clinically approved recombinant humanized mAb,^[^ ^14^ ^]^ was selected to target with high affinity its antigen, the human epidermal growth factor receptor 2 (HER2)^[^ ^15^ ^]^ which is overexpressed in 20–30% of invasive breast and ovarian carcinomas.^[^ ^16^ ^]^ We investigated the advantage of targeted immunoliposomes, obtained by decorating the surface of liposomes with Fab’ moieties coupled to a PEG spacer bearing a phospholipid moiety for the anchoring to the phospholipid bilayer (**Figure** **1**A). Given better understanding of the role of Fab’ targeting for liposomes and the relevance of the stable anchoring of the targeting complex (Fab’‐PEG) on the surface of liposomes, which commonly relies on the hydrophobic interaction of a single 1,2‐distearoyl‐sn‐glycero‐3‐phosphoethanolamine (DSPE) with the phospholipid bilayer, we also investigated immunoliposomes having a PEG shielding of improved stability. We previously studied super stealth liposomes (SSLs, Figure 1A) in which the common PEG‐DSPE, used for shielding the liposomes, was replaced with PEG derivatives carrying 2 or 4 DSPE molecules, thus allowing enhancing the hydrophobic interaction with the liposome bilayer.^[^ ^17^, ^18^, ^19^ ^]^ SSLs proved increased stability, prolonged elimination half‐life, and lower uptake by reticuloendothelial system organs in comparison with classic stealth liposomes (SLs) prepared with PEG‐DSPE. We postulated that increased stability on the PEG shielding can be beneficial also for immunoliposomes, not only for ensuring a permanent PEG attachment on the surface of the liposomes but also for achieving the same result for the PEG chains bearing the Fab’ (i.e., Fab’‐PEG‐(DSPE)2). This modification might warrant a stable anchoring of the Fab’ moieties on the surface of liposomes in an in vivo context, generating a class of SSIL2.

Furthermore, the use of Fab’ moieties, coupled through their Cys residues originally located in the hinge region of the mAb, offered the possibility of a homogenous attachment of PEG chains in the same position of the protein thus achieving a better orientation of the Fab’ over the surface of the liposomes, while leaving the Fab’ areas dedicated to antigen recognition (namely the CDR) free. Common amino coupling strategies used for full mAb conjugation would generate non‐homogenous derivatives, potentially compromising the mAb's orientation and ability to recognize the antigen, especially when the mAb is coupled close to the CDR.

Here, three generations of doxorubicin (DXR)‐loaded liposomes have been compared in an in vitro / in vivo study, namely: 1) non‐targeted SL, 2) TRZ‐targeted SIL, and 3) TRZ‐targeted SSIL2. DXR is a widely used anticancer drug either in the free form or encapsulated in liposomes, like Doxil.^[^ ^20^ ^]^ The liposomes were first tested in vitro against HER2+ (SKOV3, BT474, SKBR3) and HER2‐ (MDA‐MB‐231) cell lines, evaluating their activity and internalization, and then in vivo in different model organisms: 1) to confirm their toxicity and tumor targeting properties in a zebrafish‐based setup; 2) to assess their pharmacokinetic and toxicological profile in rats; 3) to evaluate their antitumor activity in an immunodeficient mouse xenografted with HER2‐positive ovarian cancer cells. Overall, the results presented suggest that SSIL2 represents a forward step toward an efficient targeted anticancer therapy with reduced off‐target adverse effects.

## 2 Experimental Section

### 2.1 Materials

PEG5kDa‐NHS and Boc‐PEG5kDa‐NHS were purchased from Iris Biotech GmbH (Marktredwitz, Germany); MAL‐PEG5kDa‐DSPE, hydrogenated soybean phosphatidylcholine (HSPC), and DSPE from NOF Corporation (Tokyo, Japan). *N*‐(β‐maleimidopropyloxy)succinimide ester (BMPS) was acquired from Proteochem (Loves Park, IL, USA). Doxorubicin (DXR) was provided by 21CEC PX Pharm Ltd. (Eastbourne, East Sussex, UK), and TRZ as a commercial formulation Herceptin was acquired from the pharmacy distribution. All chemical reagents, including salts and solvents, cholesterol (CHOL), micro‐BCA and BCA Protein Assay Kit for protein quantification and ProteoSilver Silver Stain Kit for protein visualization, sepharose CL‐4B cross‐linked resin, Amicon Ultra‐15 centrifugal devices were purchased from Merck Life Science s.r.l. (Milan, Italy). Precast gels for SDS‐PAGE 4–15% were from Bio‐Rad (Milan, Italy). PD‐10 Desalting Columns with Sephadex G‐25 resin and Pierce Protein Concentrators (PES) were from Thermo Fisher Scientific (Waltham, MA, USA). Female Sprague Dawley rats for pharmacokinetics and CB17/lcr‐PRKdcscid/lcrcoCrl female mice for efficacy studies were obtained from Charles River Laboratories International (Wilmington, MA, USA).

Zebrafish (Danio rerio) experiments were performed with the wild‐type line “Giotto”^[^ ^21^ ^]^ and the albino line “casper” (ZFIN ID: ZDB‐FISH‐180503‐28).

All reagents and disposable materials (Falcon) for cell culture were supplied by Corning (Corning Incorporated, NY, USA). ATPlite Luminescence ATP Detection Assay System was from PerkinElmer (Waltham, MA, USA).

### 2.2 Synthesis of PEG‐DSPE Derivatives

PEG‐phospholipid derivatives were synthesized in‐house starting either from commercial PEG5kDa‐NHS or Boc‐NH‐PEG5kDa‐NHS, by derivatization with β‐glutamic acid and coupling to two molecules of DSPE, as previously described.^[^ ^17^ ^]^ Briefly, β‐Glutamic acid (βGlu, 147.13 Da, 3 eq.) was conjugated to the activated polymers in acetonitrile / 0.1 m borate buffer pH 8.0 (2:3 v/v) and the intermediates were purified by extraction with dichloromethane and precipitation in cold diethyl ether. The carboxyl groups of βGlu in PEG5kDa‐βGlu or Boc‐NH‐PEG5kDa‐βGlu were then activated by *N*,*N*′‐dicyclohexylcarbodiimide (DCC, 206.33 Da, 3 eq.) and *N*‐hydroxysuccinimide (NHS, 115.09 Da, 1.5 eq.), yielding PEG5kDa‐βGlu(NHS)_2_ or Boc‐NH‐PEG5kDa‐βGlu(NHS)_2_. DSPE (1.2 fold molar excess with respect to NHS groups of intermediates) was coupled to PEG5kDa‐βGlu(NHS)_2_ or Boc‐NH‐PEG5kDa‐βGlu(NHS)_2_ in chloroform at 50 °C for 6 h. The excess of DSPE was removed with lauroyl chloride in chloroform and the product was recovered by precipitation in cold diethyl ether. The powder was solubilized in 20% MeOH and purified by dialysis against 20% MeOH (3.5 kDa MWCO membranes) and then water. After lyophilization, the purified products were characterized by 1H‐NMR and by 1H‐DOSY NMR spectroscopy using a Brüker Avance 400 spectrometer (Rheinstetten, Germany) operating at 400 MHz.

Boc‐NH‐PEG5kDa‐(DSPE)_2_ derivative was subsequently dissolved in dichloromethane / TFA (50:50 v/v) at a final PEG concentration of 2% w/v. The solution was stirred for 20 min at 4 °C to remove Boc protecting group. The solvent was removed under vacuum and the product was recovered by precipitation in cold diethyl ether. 1H‐NMR spectroscopy confirmed the removal of the Boc group. The intermediate was then reacted in 0.1 m phosphate buffer pH 7.2 / ACN 3:1 v/v with N‐(β‐maleimidopropyloxy)succinimide ester (BMPS, 266.21 Da, 8 eq.), previously dissolved in DMSO. After 45 min, the reaction mixture was dialyzed against water (14 kDa MWCO membrane) and the resulting MAL‐PEG5kDa‐(DSPE)_2_ derivative was characterized by ^1^H‐NMR and ^1^H‐DOSY NMR spectroscopy.

### 2.3 Preparation of TRZ Fab’ and Coupling to PEG‐DSPE Derivatives

TRZ was enzymatically digested with pepsin (E/S 1:50 w/w) for 3 h at 37 °C in 0.1 m acetate buffer pH 3.8 yielding the F(ab’)_2_ fragment, which was purified by gel filtration on a Superose 12 10/300 GL column (GE Healthcare, USA) equilibrated in PBS pH 7.4 at 0.5 mL min^−1^ flow‐rate. The purification was performed using an AKTA purifier (GE Healthcare, Uppsala, Sweden), monitoring at 280 nm. The eluted F(ab’)_2_ was concentrated by ultrafiltration at 10 mg mL^−1^ and then reduced with 10 mm cysteamine (113.61 Da) for 30 min at room temperature, yielding Fab’ fragments. Fab’ was purified by filtration on a Superdex 200 Increase 10/300 GL column (GE Healthcare, Uppsala, Sweden) eluted with PBS pH 7.2, 10 mm EDTA at 0.5 mL min^−1^ flow‐rate. The pool Fab’ fraction was reacted with MAL‐PEG5kDa‐DSPE or MAL‐PEG5kDa‐(DSPE)_2_ derivative (tenfold molar excess) and the solution was left at room temperature overnight. Unreacted maleimide groups of PEG‐DSPE derivatives were blocked by reaction with twofold molar excess of thioglycolic acid (15 min, room temperature).

Proteins quantification was performed by BCA assay or UV–vis absorption at 280 nm (A^0.1%^_280_TRZ = 1.43; A^0.1%^_280_Fab’_TRZ_ = 1.35). Additionally, the intermediates and the final product were characterized by SDS‐PAGE and MALDI‐TOF mass spectrometry (Xevo G2‐S Q‐Tof instrument, Waters Corporation—Milford, MA, USA).

### 2.4 Formulation of SL, SIL, and SSIL2

Non‐targeted SL and SSL2 formulations were prepared as previously described.^[^ ^17^ ^]^ Fab’_TRZ_‐coupled PEG‐lipid(s) derivatives were transferred into pre‐formed DXR‐loaded SL or SSL2 by post‐insertion^[^ ^22^ ^]^ to obtain the corresponding Fab’_TRZ_‐targeted SIL and SSIL2. Briefly, empty PEGylated liposomes were prepared by thin layer evaporation using a lipid mixture composed of HSPC : CHOL : PEG5kDa‐DSPE or PEG5kDa‐DSPE_2_ (16:8:1 molar ratio). The dried lipid film was rehydrated (1 h at 60 °C) with 250 mm ammonium sulfate pH 5.0 and the suspension was homogenized by freeze‐thaw and hot extrusion at 60 °C through 200‐100‐50 nm polycarbonate filters (Whatman Nuclepore Track‐Etched Membranes) by using LiposoFast Basic extruder (Avestin Europe GmbH‐Manheim, Germany). The obtained PEGylated liposomes were purified on a PD‐10 desalting column eluted with PBS pH 7.4. DXR was encapsulated by remote loading. A solution of empty SL or SSL2, at the concentration of 10 mg mL^−1^ HSPC equiv., was incubated with DXR (5 mg mL^−1^) for 1 h at 60 °C. HSPC concentration was assessed by Stewart Assay.^[^ ^23^ ^]^ Loaded DXR was quantified by UV–vis absorption at 477 nm (*ℇ*_477_ = 13 050 m ^−1^ × cm^−1^) upon vesicles disruption with 5% v/v 1 m Triton X‐100.

The corresponding non‐targeted DXR‐loaded SL or SSL2 were thereafter incubated for 1 h at 60 °C (400 rpm) with a 2 mol% PEG micelles solution composed of PEG5kDa‐DSPE : Fab’‐PEG5kDa‐DSPE (molar ratio 3 : 1) or PEG5kDa‐(DSPE)_2_ : Fab’‐PEG5kDa‐(DSPE)_2_ (molar ratio 1 : 1), respectively, to achieve a comparable Fab’ density on the surface of both formulations. Similarly, the non‐targeted SL were incubated with 2 mol% w/v micelles solution of PEG5kDa‐DSPE to obtain the reference suspension of SL with an equal amount of PEG compared to SIL and SSIL2. All the suspensions were then eluted on a Sepharose CL‐4B column, and equilibrated in PBS pH 7.4, to purify SL, SIL, and SSIL2. Fab’ constructs decoration of liposomes was assessed by SDS‐PAGE analysis. Micro‐BCA assay was used to quantify the amount of Fab’ on liposomes surface.

### 2.5 Physico‐Chemical Characterization of Liposomes

Targeted and non‐targeted liposomes were characterized by dynamic light scattering (DLS) to evaluate particles’ mean size, polydispersity index (PDI), and zeta‐potential (ζ) using a Zetasizer Nano ZS (Malvern Instrument Ltd., Worcestershire, UK). Liposome morphology and homogeneity were assessed by transmission electron microscopy (TEM). One drop of liposomal suspension (about 25 µL) was placed on 400 mesh holey film grid. The samples were diluted with deionized water to avoid as much as possible salts interference. After staining with 1% uranyl acetate the sample was observed with a Tecnai G2 (FEI) transmission electron microscope operating at 100 kV. Images were captured with a Veleta (Olympus Soft Imaging System) digital camera. Additionally, SIL and SSIL2 were immunolabeled with IgG‐gold nanoparticles to evaluate the presence of Fab’ fragments on the bilayer compared to non‐targeted SL (negative control). Briefly, one drop of liposomes was placed on specimen grids and blocked with 0.5% BSA in PBS (10 min), followed by incubation with 10–20 µL of a primary mouse monoclonal anti‐TRZ F(ab’)_2_ antibody (#TRB‐Y1, Acrobiosystems, Newark, DE, USA) at 0.4 µg mL^−1^ (30 min). The grids were then washed with PBS (3× 2 min) and 10–20 µL of secondary anti‐mouse antibody (1:30), conjugated with 5‐nm gold nanoparticles, were added. After 30 min of incubation, the grids were washed with PBS (2× 2 min) and then with water (3× 1 min). Following, the samples were stained with 0.5% uranyl acetate and subsequently with 1% Pb citrate, and images were captured as previously described.

### 2.6 Long‐Term Stability Studies

300 µL of each liposomal formulation (3 mm in HSPC), containing 0.05% NaN_3_ w/v, were incubated for 2 months at 4 and 25 °C. DLS measurements were performed at predetermined time points to assess the vesicle mean size and PDI and, thus, evaluate the overall physical stability of the formulation over time.

### 2.7 Drug Release Studies

Doxorubicin release from liposomal formulations was investigated by the fluorescence dequenching of self‐associated DXR in liposomes upon dilution outside the liposomes.^[^ ^24^ ^]^ Briefly, targeted and non‐targeted liposomes (1 mm in HPSC, 500 µL) were incubated for 16 h at 37 °C in PBS and the fluorescence intensity associated to DXR was measured every 30 min, using a Jasco FP‐6500 spectrofluorometer (*λ*_ex_ = 470 nm; *λ*_em_ = 584 nm). At the end of each analysis, liposomes were disrupted by the addition of 5% v/v of 1 m Triton X‐100 to evaluate the maximum fluorescence of dequenched DXR (total DXR).

### 2.8 Cell Culture and Coculture

For cytotoxicity experiments, four human cancer cell lines were used, that is, three HER2+ BT474 (ATCC HTB‐20), SKBR3 (ATCC HTB‐ 30) and SKOV3 (ATCC HTB‐77), and the HER2‐ MDA‐MB‐231 (ATCC HTB‐26). Cells were maintained in DMEM medium supplemented with 10% fetal bovine serum (FBS), 1% glutamine, 1% penicillin‐streptomycin, in humidified conditions at 37 °C with 5% CO_2_.

A coculture model was set up, using THP‐1 cells (ATCC TIB‐202), differentiated into MΦ macrophages, and SKOV3 cells, to evaluate the effect of SL, SIL, and SSIL2 in the differentiation of macrophages to protumoral tumor‐associated macrophages (TAMs). To this aim, transwells with 0.8 µm pore‐membranes (Falcon, Corning Incorporated, NY, USA) suitable for the 24‐well plate were used, on which SKOV3 cells were seeded. THP1 cells were seeded on coverslips, with a density of 105 cells/pc, diluted in RPMI medium, and treated for 24 h with 320 nm of phorbol 12‐myristate 13‐acetate (PMA, Santa Cruz Biotechnology, Inc., Santa Cruz, CA, USA), to allow their differentiation into MΦ macrophages. After 24 h, the transwells were placed in the wells containing the THP‐1‐derived MΦ macrophages, and the liposomal SL and immunoliposomal SIL and SSIL2 formulations were added at the IC50 concentration. After 24 h, immunocytochemistry was performed to assess the expression of TAM markers.

### 2.9 In Vitro Cytotoxicity Studies

The cytotoxic effect of formulations was evaluated on HER2+ (BT474, SKBR3, and SKOV3) and HER2‐ (MDA‐MB‐231) cell lines by means of the ATPlite Luminescence ATP Detection Assay System. The cytotoxic effect of targeted DXR‐loaded liposomal formulations was compared to that of non‐targeted DXR‐loaded liposomes and free DXR (positive control). Cells treated only with the medium were used as control. Briefly, the cells were seeded in black 96‐well plates at a density of 5000 cells/well (SKBR3, SKOV3, and MDA‐MB‐231) and 7500 cells/well (BT474). After 24 h, cells were treated with increasing concentrations of the liposomal formulations (0.01–50 µm in DXR, *n* = 7) for 24 (SKBR3, SKOV3, and MDA‐MB‐231) or 48 (BT474) h. At the end of the exposure, treatments were replaced by fresh complete medium for 24 h. To quantify ATP production, cells were treated following the manufacturer's instructions, and luminescence was quantified by means of a Victor Nivo multi‐plate reader (PerkinElmer, Waltham, MA, USA). The percentage of cell viability was determined as (luminescence_treated cells_/luminescence_control cells_) × 100. IC50 values were calculated using GraphPad Prism 9.0 (San Diego, CA, USA) by a nonlinear regression model.

### 2.10 Evaluation of DXR Internalization and Nuclear Localization

SKBR3 cells were seeded into 24‐well plates with a glass coverslip (3 × 10^4^ cells/well). After 24 h, cells were treated with formulations (5 µm in DXR) or free DXR (0.02 µm) for 1, 2, 4, 6 and 24 h. A control well, containing cells incubated only with medium, was used to exclude cell autofluorescence. At the end of exposure, cells were washed twice with PBS and fixed for 30 min with 4% PFA.^[^ ^25^ ^]^ After an additional PBS wash, cells were incubated with Hoechst 33342 to counterstain nuclei and mounted with Mowiol. The images were acquired using a confocal microscope Zeiss LSM 800 (Milan, Italy). In order to compare the different formulations, ImageJ software was used to quantify the intensity of the DXR internalized at different time points and to assess the colocalization with the nuclear marker Hoechst 33342.

### 2.11 Immunofluorescence Coupled to Confocal Microscopy

This technique was used to assess the membrane expression of HER2 in SKOV3, SKBR3, and BT474 cells, and the TAM phenotype in THP‐1 cells co‐cultured with SKOV3 by measuring prototypical markers, that is, the protein expression of CD68 and CD163. Immunocytochemistry was performed as previously described by using an anti‐HER2 antibody (Santa Cruz Biotechnology, Inc.), an anti‐CD68 (Abcam, Cambridge, UK), and an anti‐CD163 primary antibody (Santa Cruz, CA, USA).^[^ ^26^ ^]^ Images were acquired at 63× magnification with a T‐lapse Zeiss LSM800 microscope and then analyzed with ImageJ software.

### 2.12 Live‐Cells Confocal Microscopy

To assess the extent of liposome uptake and internalization, a live confocal microscopy experiment was performed using green fluorescent SKOV3 cells (LINTERNA SK‐OV‐3, Innoprot, Bizkaia, Spain), maintained in McCoy medium supplemented with 10% FBS, 0.5% l‐glutamine, and 0.5% G418. For the experiment, 35 × 10^4^ cells were seeded in 4‐well glass‐bottom culture chambers (Ibidi GmbH, Germany) and loaded with the fluorescent DiD‐stained SL and SSIL2. Images were periodically acquired for 24 hours using a time‐lapse confocal microscope (Zeiss LSM 800, 40× magnification).

### 2.13 Evaluation of DXR Cellular Uptake in a Competition Assay with Trastuzumab

SKOV‐3 cells were seeded in a 24‐well plate (10^5^ cell in 1 mL) and, after 24 h, incubated with trastuzumab (140 nm) dissolved in complete medium for 2 h at 37 °C. Then, cells were treated with liposomes (SSL2 or SSIL2) at a concentration of 5 mm in DXR. After 24 h, the medium was removed, and cells were trypsinized and centrifuged to obtained single cell suspension. Cells treated with complete medium were used as controls. DXR uptake was assessed by evaluating the number of DXR‐positive cells by flow cytometry (BD FACSAria III, 10000 events/sample, ex/em 460/570 nm).

### 2.14 Evaluation of DXR Cellular Uptake in Presence of Protein Corona

Liposomes (SL, SIL, SSL2, and SSIL2, 100 mg in HSPC) were incubated in 1 mL of a solution containing rat EDTA‐treated plasma (10% and 55% in PBS) for 2 h at 37 °C with gentle shaking, to allow the formation of protein corona. To collect corona‐forming proteins, liposomes were centrifuged for 20 min at 15 000 × *g* at 4 °C. SKOV‐3 were treated with liposomes, either preincubated or not with rat plasma, at 5 mm DXR. After 24 h, the medium was removed, and cells were trypsinized and centrifuged to obtained a single cell suspension. Cells treated with complete medium were used as negative controls. DXR uptake was assessed by evaluating the number of DXR‐positive cells by means of flow cytometry (BD FACSAria III, 10000 events/sample, ex/em 460/570 nm).

### 2.15 Pharmacokinetic and Toxicological Studies

The study protocol was approved by the Ethics Committee of the University of Padova and the Italian Ministry of Health (Prot. No. 938/2016‐PR obtained on October 10, 2016), and animals were handled in compliance with national and international guidelines, that is, the Italian Legislative Decree 26/2014 and the “Guide for the Care and Use of Laboratory Animals” by the National Research Council of the National Academies. Female Sprague Dawley rats (140–190 g) were randomly divided into groups of 6 rats each. A dose of 2.5 mg kg^−1^ in DXR of either free drug or DXR‐loaded liposomal formulations (SL, SIL, SSIL2) was administered via tail vein to the rats previously anesthetized with isoflurane gas (mixed with O_2_ in enclosed cages). At scheduled time points, blood samples (≈200 µL) were collected from the tail into heparin‐treated tubes and thereafter immediately centrifuged 15 min × 1500 × *g* to separate plasma. Rats were euthanized with isoflurane anesthesia 48 h after administration and the blood was collected with an intracardiac puncture to measure complete blood count and plasma biochemistry. These measurements were performed by means of standard laboratory tests.^[^ ^27^ ^]^ After sacrifice, the main organs (liver, spleen, lungs, kidneys, heart, brain, ovaries) were collected, weighed, washed with saline solution, and frozen in liquid nitrogen. An appropriate amount of each organ was placed in formalin for histological analysis.^[^ ^28^ ^]^

DXR was extracted by treating 50 µL of plasma with 10 µL of 1 m Triton X‐100 and 580 µL of 81 mm HCl in isopropanol to allow plasma proteins precipitation. After overnight incubation at 4 °C, samples were centrifuged for 3 min × 3000 rpm and the supernatants were analyzed using an FP‐6500 Jasco spectrofluorometer (*λ*_ex_ = 470 nm; *λ*_em_ = 584 nm). DXR concentration in each plasma sample was extrapolated through a calibration curve of standard solutions of either DXR or DXR‐loaded SL for the liposomal formulations. The pharmacokinetic analysis was performed using the PKSolver 2.0 software by applying a bicompartmental model.

### 2.16 Quantification of Gene Expression in Rat Tissue

RNA from liver and spleen tissues was extracted by means of a total SV RNA isolation kit (Promega Corporation, Madison, WI), as described in detail previously.^[^ ^27^ ^]^ The relative mRNA expression of *Il6*, *Il1b*, *Tnfa*, *Il10*, *Ccl2*, and was measured by qRT‐PCR using the commercial kit One Step SYBR Prime Script RT‐PCR (Takara, Mountain View, CA, USA) and calculated according to the ∆∆Ct method, as previously described.^[^ ^29^ ^]^

The primers used in this study are listed in **Table** **1**.

**Table 1:** Primers used in the qRT‐PCR analysis.

Gene	Sequence forward 5′–3′	Sequence reverse 5′–3′
*Il6*	gac aaa gcc aga gtc att cag	gtc ctt agc cac tcc ttc t
*Il1b*	aaa tgc ctc gtc tgt ctg a	caa ggc cac agg gat ttt gtc
*Tnf*	gat cgg tcc caa caa gga gg	gct ggt ggt ttg cta cga c
*Il10*	taa aag caa ggc agt gga gc	tgc cgg gtg gtt caa ttt ttc
*Ccl2*	tga tcc caa tga gtc ggc tg	tgg acc cat tcc tta ttg ggg
*Cxcl2*	tgc tca aga ctc caa cca ctc	cca caa caa ccc ctg tac cc
*Actb*	gcc acc agt tcg cca tgg a	ttc tga ccc ata ccc acc at

### 2.17 In Vivo Efficacy Studies in Zebrafish and Mouse Models

Zebrafish. Zebrafish embryos and adults were raised, staged, and maintained at the DeBio Zebrafish Facility of the University of Padova, under standard conditions.^[^ ^30^ ^]^ Zebrafish experiments were performed in accordance with the Italian and European Legislations (Directive 2010/63/EU) and with permission for animal experimentation from the Ethics Committee of the University of Padova (OPBA) and the Italian Ministry of Health (Auth. no. 407/2015‐PR).

For anesthesia or euthanasia of zebrafish embryos and larvae, Tricaine (E10521, Sigma‐Aldrich, St. Louis, MO, USA) was added to the fish water at 0.16 mg mL^−1^ or 0.3 mg mL^−1^, respectively.

For xenotransplantation, embryos were mechanically dechorionated at 2 days post‐fertilization (dpf), Tricaine‐anesthetized, and placed along plastic lanes immersed in 2% methylcellulose/PBS. SKOV3 cells were resuspended in 10 µL PBS at a density of 1 × 10^5^ cells µL^−1^, loaded in a glass capillary needle and microinjected in the anterior part of the yolk (about 100 cells/embryo), using a WPI PicoPump apparatus. Liposome formulations (5 µm concentration in DXR) were separately microinjected (5 nL/embryo) in the posterior part of the yolk. Xenotransplanted embryos were grown at 33 °C and monitored daily, removing dead individuals. At 1 day post‐injection (dpi), zebrafish displaying cells in the circulatory system, likely due to microinjection damage, were discarded.

Daily observations were performed using a Leica M165FC dissecting microscope equipped with a Leica DFC7000T camera. Confocal imaging was made with Nikon C2 and Leica SP5 systems on anesthetized individuals, mounted in 1% low melting agarose gel. Fluorescent intensities from SKOV3 cells and liposomes were counted and quantified on identical ROIs (regions of interest) using the Measurement option of the Volocity 6.0 software (PerkinElmer, Milan, Italy). Colocalization signals were evaluated by counting liposome/cell fusions and by global Pearson's R correlation analysis.

### 2.18 Mice

The anticancer activity of SL, SIL, and SSIL2 was also evaluated in vivo in immunodeficient female mice (CB17/lcr‐PRKdc^scid^/lcrcoCrl mice) xenografted with SKOV3 cells. For the model setup, SKOV3 cells (2 × 10^6^) were mixed in sterile PBS and Matrigel (4 mg mL^−1^, Corning), and 200 µL of the suspension was inoculated subcutaneously in 6–8‐week‐old female SCID mice on the left flank. Tumor growth was monitored three times a week by a digital caliper (Tack Life, Digital Caliper), and the volume was calculated with the standard formula:

(1) $$\mathrm{Volume}\;\left({\mathrm{mm}}^{3}\right)=\frac{\left({\mathrm{length}\times \mathrm{width}}^{2}\right)}{2}$$

When the tumor volume was 85 mm^3^, mice were randomized into four groups (*n* = at least 5 mice per group). Mice of the control group received vehicle (PBS), while the three treated groups received respectively SL, SIL, or SSIL2, at the dose of 5 or 10 mg kg^−1^ (DXR equiv.), by 3 intravenous (i.v.) injections every 7 days.

Mice were sacrificed when the experimental or humane endpoint were reached and tumors was extracted and used for flow cytometry analyses.

### 2.19 Flow Cytometry

After the sacrifice, tumors were extracted and processed to obtain a single‐cell suspension. Briefly, 0.5 mg of tumors were harvested and incubated with a solution of 400 U mL^−1^ collagenase D (Merck) and 0.005 µg mL^−1^ DNAse for 1 h at 37 °C to allow the enzymatic digestion of the tissue. The cell suspension was filtered using first a 100 µm‐ and then a 40 µm‐cell strainer, centrifuged twice using 5 mL of Red Blood Cells Lysis solution (155 mm NH_4_Cl, 12 mm NaHCO_3_, 0.1 mm EDTA) at 300 RCF for 5 min to lyse red blood cells. For the detection of the intracellular antigen CD206, cells were permeabilized with 0.1% of Tween 20. 1 × 10^6^ cells mL^−1^ were incubated with anti‐CD16/32 (2.4G2, BioXCell, Lebanon, NH, USA) for 15 min at room temperature to avoid unspecific binding of the antibodies.

TAMs were identified using the following anti‐mouse antibodies: Alexa Fluor 700‐conjugated CD45, PE‐conjugated CD20 (both from eBioscience, Thermo Fisher Scientific, Waltham, MA, USA), APC‐conjugated F4/80 (BioRad, Milan, Italy).

Cells were incubated with the primary antibodies for 15 min at room temperature and then fixed with 4% formalin (Diapath SPA, Bergamo, Italy). Analyses were performed with BD FACSymphony, and data were analyzed using FlowJo v.10.0.8 software.

### 2.20 Statistical Analysis

The obtained data were analyzed with the software GraphPad Prism 9.0 and compared using one‐way ANOVA or the non‐parametric Kruskal–Wallis test when the normal distribution of data could not be demonstrated. In the event of significant differences (*α* = 0.05), the ANOVA was followed up by Tukey's posthoc test for multiple comparisons. A *p*‐value <0.05 was considered statistically significant. Unless otherwise stated, data are presented as mean ± S.D.

## 3 Results and Discussion

### 3.1 Synthesis of PEG‐bi‐Phospholipids Derivatives and Fab’ Coupling

The design of PEG‐bi‐phospholipids derivatives has proved to improve the biopharmaceutical and pharmacokinetic features of conventional stealth liposomes.^[^ ^17^, ^18^, ^19^ ^]^ Hence, to further ameliorate the system and promote the specific accumulation of such SSLs in tumors, we investigated the development of ligand‐targeted SSLs by coupling the Fab’ fragment of TRZ to the distal end of these PEG‐bi‐phospholipids. The resulting Fab’_TRZ_‐targeted SSLs are aimed to selectively recognize the HER2 receptors, over‐expressed on the surface of several cancer cells.

The synthesized PEG‐bi‐phospholipid(s) compounds were characterized by 1H‐NMR spectroscopy showing the expected ratio between the polymer and the attached phospholipid units, and 1H‐DOSY NMR spectroscopy confirmed the purity of the products (Figures S1–S3, Supporting Information). The Fab’ fragment of TRZ was obtained by proteolytic digestion of the whole monoclonal antibody, yielding the F(ab’)_2_ fragment, followed by a mild reduction step to selectively reduce the interchain disulfide bonds at the hinge region. Both F(ab’)_2_ and Fab’ were purified by size exclusion chromatography (Figure S4A, Supporting Information) and analyzed by SDS‐PAGE (Figure S4B, Supporting Information) and MALDI‐TOF analysis (Figure S5, Supporting Information) showing the expected molecular weights. Fab’ of TRZ was reacted with MAL‐PEG5kDa‐DSPE or MAL‐PEG5kDa‐(DSPE)_2_ to form the respective Fab’‐PEG‐(DSPE)*_n_* derivatives that were characterized by SDS‐PAGE (Figure S6, Supporting Information).

### 3.2 SIL and SSIL2 Characterization

Fab’‐PEG5kDa‐DSPE and Fab’‐PEG5kDa‐(DSPE)_2_ derivatives were transferred to DXR‐loaded pre‐formed SIL or SSIL2, prepared with PEG5kDa‐DSPE and PEG5kDa‐(DSPE)_2_, respectively. This process depends on a thermodynamically favorable mechanism driven by hydrophobic forces between lipid components, in a temperature‐ and time‐dependent manner.^[^ ^22^ ^]^ Gel chromatography on Superose CL‐4B column was used to purify immunoliposomes from free Fab’, non‐transferred micelles, and F(ab’)_2_ as assessed by SDS‐PAGE analysis (Figure S7, Supporting Information). The Fab’‐targeted liposomal formulations were characterized and compared to non‐targeted formulations. According to DLS measurements, all suspensions were homogeneous (PDI < 0.1), with a mean particle size in the range of 85–100 nm and a slightly negative surface potential (ζ) (**Table** **2**). The TEM images confirmed the DLS analysis (Figure 1B). The amount of ligand incorporated within the liposome bilayer ranged between 5–7 µg Fab’/µmole HSPC as quantified by micro‐BCA assay. The theoretical number of Fab’ fragments per liposome was calculated according to literature,^[^ ^31^ ^]^ assuming that liposomes were spherical unilamellar vesicles of 100 nm in diameter, composed of HSPC:CHOL (molar ratio 2:1), and estimating that 1 µmole of HSPC forms 7.8 × 10^12^ liposomes. Since 1 µg of protein corresponds to 1.2 × 10^13^ Fab’, it was calculated that the average Fab’/liposome was 9–11 for both SIL and SSIL2. In accordance with these findings, the presence of multiple Fab’ on the immunoliposomes surface was confirmed by the TEM images acquired after immunolabelling (Figure 1C).

**Table 2:** Physicochemical characterization by dynamic light scattering (DLS) analysis of the liposomal formulations.

Formulation	Size [nm]	PDI	Zeta‐potential
empty‐SL	90.62 ± 0.91	0.036 ± 0.017	−2.52 ± 0.40
empty‐SSL2	87.31 ± 2.13	0.032 ± 0.016	−1.43 ± 0.40
SL‐DXR	93.22 ± 4.22	0.056 ± 0.025	−4.08 ± 0.61
SSL2‐DXR	92.68 ± 5.62	0.056 ± 0.023	−2.88 ± 0.60
SIL‐DXR	97.01 ± 0.68	0.037 ± 0.018	−3.64 ± 0.91
SSIL2‐DXR	88.69 ± 0.78	0.060 ± 0.016	−2.41 ± 0.36

Data are expressed as mean ± S.D. and are the results of at least three independent experiments.

The stability of all liposomal formulations was monitored for 2 months by incubating them at 4 and 25 °C. All tested formulations were stable, preserving the same mean size and polydispersity over the time (Figure S8, Supporting Information), confirming the stabilizing effect of PEG that prevented vesicles aggregation by steric hindrance. Similarly, the liposomes showed a good stability during the time when incubated in plasma for up to 55 h (Figure S9, Supporting Information).

The DXR encapsulation efficiencies calculated for SL and SSL2, before the post insertion of the Fab’‐PEG‐(DSPE)*_n_* derivatives, were always >90%. For targeted immunoliposomes, it is important that the drug is not released before the vesicle is internalized inside the targeted cell, a process that should be selective due to the HER2 targeting achieved by the Fab’ of TRZ. The drug is stably entrapped inside the liposomes as shown by the absence of drug release over 16 h incubation at 37 °C (Figure S10, Supporting Information). Thus, the inclusion by post‐insertion of mixed micelles composed of PEG‐(DSPE)*_n_* and Fab’‐PEG‐(DSPE)*_n_* into DXR‐loaded pre‐formed SL and SSL2 did not affect the integrity of the phospholipid bilayer. The release of DXR is slightly detectable when the liposomes were incubated in buffer at pH 5.5 (37 °C), but still after 16 h the maximum release was lower than 1% (Figure S11, Supporting Information).

### 3.3 In Vitro Evaluation of Anticancer Activity

**Figure 2:**
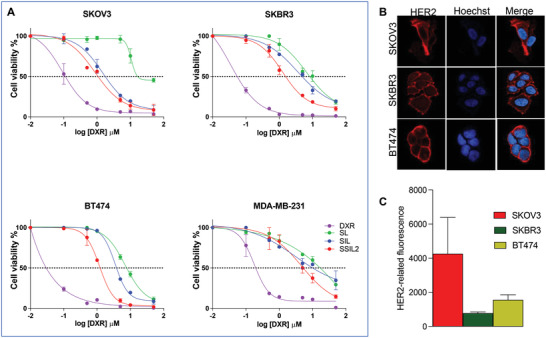
In vitro evaluation of the anticancer activity. A) In vitro concentration‐response curves of SKOV3 (HER2+), SKBR3 (HER2+), BT474 (HER2+), and MDA‐MB‐231 (HER2‐) treated with DXR‐loaded targeted (SIL and SSIL2) and non‐targeted (SL) liposomes. B) Expression of HER2 in the plasma membrane of HER2+ cells, and C) quantification of HER2‐related fluorescence per cell.

As expected, DXR exhibited in both HER2+ (SKBR3, BT474, SKOV3) and HER2‐ (MDA‐MB‐231) cell lines the greatest antiproliferative effect (**Figure** **2**), due to its low molecular weight and amphiphilic nature, allowing a fast and effective diffusion across cell membranes. The calculated IC50 values for DXR were 0.080 ± 0.003  µm, 0.034 ± 0.026 µm, 0.092 ± 0.004 µm, and 0.287 ± 0.002 µm for BT474, SKBR3, SKOV3, and MDA‐MB‐231, respectively. As expected, these values were significantly lower than the IC50 calculated for all liposomal formulations in each cell line (*p* < 0.001).

In HER2+ cell lines, SIL‐DXR and SSIL2‐DXR displayed a higher cytotoxic activity with respect to non‐targeted SL‐DXR at the tested conditions, thereby evidencing the targeting effect of the Fab’ fragment of TRZ (Figure 2A). The effect of targeting was particularly evident in SKOV3 cells in our experimental conditions, in accordance with the higher expression of the HER2 antigen in this cell line, when compared to SKBR3 and BT474 cells (Figure 2B,C). Ligand‐receptor interaction is known to elicit receptor‐mediated endocytosis of intact liposomes thus enhancing the intracellular amount of the drug and consequently the cytotoxic effect in vitro, provided that the encapsulated drug is released from the endosome‐lysosome compartment.^[^ ^32^, ^33^ ^]^ Furthermore, SSIL2‐DXR resulted to be the most potent formulation in inducing cell death, being their IC50 values significantly lower than those of SL‐DXR and SIL‐DXR (**Table** **3**). This effect was particularly evident in SKBR3 cells, where we performed the experiment described in the following paragraph.

**Table 3:** IC50 values of liposomal formulations loaded with DXR in different cell lines.

Cell line	SL‐DXR [µm]	SIL‐DXR [µm]	SSIL2‐DXR [µm]
BT474	7.801 ± 1.438	3.266 ± 0.256^**^	1.119 ± 0.120^***,^ ^#^
SKBR3	7.656 ± 1.187	4.475 ± 0.429^**^	1.001 ± 0.104^****^, ^##^
SKOV3	10.65 ± 2.341	1.475 ± 0.327***	0.8703 ± 0.0971***
MDA‐MB‐231	10.512 ± 2.968	9.278 ± 1.474	7.535 ± 2.906

Data are expressed as mean ± S.D. and are the results of at least three independent experiments;

** *p* < 0.01; p<0.001

**** *p* < 0.001 versus SL‐DXR;

^#^ *p* < 0.05;

^##^ *p* < 0.01 versus SIL‐DXR; one‐way ANOVA followed by Tukey's post hoc test for multiple comparisons.

**Figure 3:**
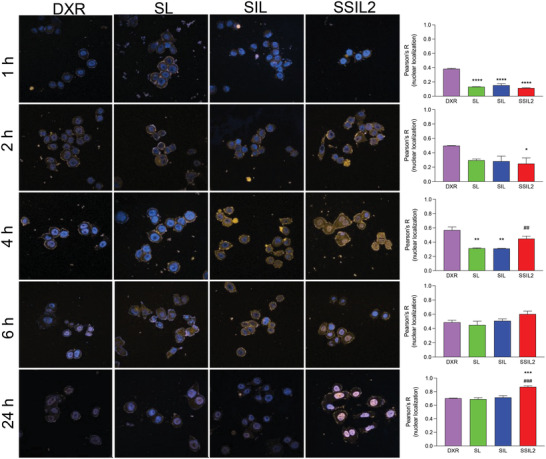
Cell internalization study. DXR internalization and nuclear localization by SKBR3 cells (HER2+) after 1 h, 2 h, 4 h, 6 h, and 24 h of treatment with the liposomal formulations (5 µm in DXR equiv.), compared to free DXR (0.02 µm). The Pearson's *R* for colocalization with the nuclear blue signal of Hoechst 33342 is reported in the graph on the right. ^*^ *p* < 0.05, ^**^ *p* < 0.01, ^***^ *p* < 0.001 versus cells treated with DXR, ^##^ *p* < 0.01, ^###^ *p* < 0.001 versus cells treated with SL or SIL.

Since the cytotoxic activity of the liposomal formulations is correlated to their endocytosis rate by the cells, confocal fluorescence microscopy was performed in the HER2+ SKBR3 cell line, to assess the kinetic of internalization of these nanocarriers within target cancer cells in vitro. As shown in **Figure** **3**, the targeting effect was not significantly evident for SIL‐DXR liposomes, since they showed a profile of DXR internalization similar to SL‐DXR. In contrast, SSIL2‐DXR displayed a significantly higher intracellular and nuclear uptake of DXR, being the DXR‐associated intracellular fluorescence significantly higher than that obtained with SL‐ and SIL‐DXR 4 h and, to a higher extent, 24 h after the beginning of cell incubation. Therefore, the presence of PEG‐bi‐phospholipids, together with the targeting effect performed by Fab’_TRZ_, significantly increases the nuclear presence of DXR in SKBR3 cells after 24 h of incubation, with the nuclear DXR significantly higher in SSIL2 treated cells than that of cells treated with SL and SIL. The increased targeting effect of SSIL2 was confirmed by live confocal imaging, showing the fastest and more effective interaction between DiD‐labelled SSIL2 (red fluorescence) and LINTERNA SK‐OV‐3 (green fluorescence), with respect to untargeted SL liposomes and SIL (Videos S1–S3, Supporting Information). The specificity of the targeting effect was confirmed by a competition study performed in SKOV3 cells, where only the internalization of DXR‐loaded in SSIL2 was reduced by 50% after preincubation of cells with TRZ, whereas the internalization of DXR‐loaded untargeted SSL2 was unaffected (Figure S12, Supporting Information).

Then, to understand the role of protein corona on the internalization of immunoliposomes by HER2‐expressing SKOV3 cells, we evaluated the effect of preincubation liposomes with rat plasma, demonstrating that, the preincubation with 55% v/v of rat plasma significantly affected DXR positivity of SKOV cells for all formulations. This effect was higher for SSIL2 and their untargeted counterpart SSL2, than that observed for SIL and SL. In fact, DXR fluorescence reduction after plasma preincubation with respect to not preincubated liposomes was 38% and 30% for SL and SIL, respectively, and of 66% and 63% for SSL2 and SSIL2, respectively (Figure S13, Supporting Information). These findings indicate that within the same liposomes couples (i.e., SL/SIL or SSL2/SSlL2) the presence of the targeting agent is not influencing protein corona formation, but the same may be affected by the different PEG layer.

### 3.4 Pharmacokinetic and In Vivo ADME‐Tox Analysis of Liposomal Formulations

**Figure 4:**
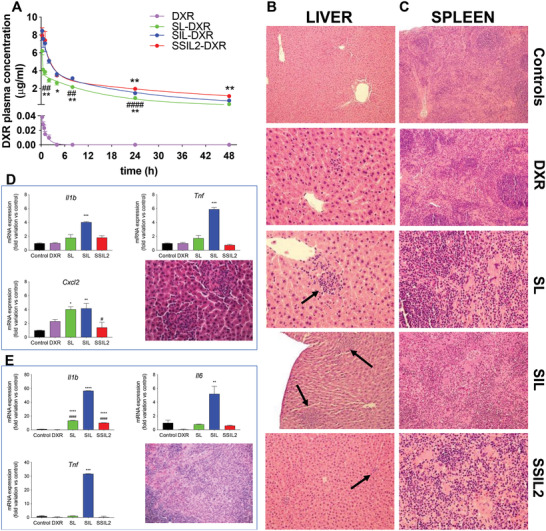
Pharmacokinetic profiles and toxicity analysis of liposomes in rats. A) In vivo pharmacokinetic profiles of free DXR, SL‐DXR, SIL‐DXR, and SSIL2‐DXR. ^*^ *p* < 0.05 and ^**^ *p* < 0.01 versus SIL‐DXR, ^##^ *p* < 0.01 and ^####^ *p* < 0.0001 versus SSIL2‐DXR. Representative photomicrographs of B) the liver and C) spleen sections of a control rat (no histological alterations could be observed in the liver and spleen); rat treated with free DXR (small and isolated granulomatous lesion in the liver and slight increased hematopoiesis in the splenic tissue could be observed); rat treated with SL–DXR and SIL–DXR (multiple granulomatous lesions in the liver, indicated by arrows, and extensive fibrosis associated with increased hematopoiesis in the splenic tissue could be observed) while for SSIL2‐DXR only small and isolated granulomatous lesions have been observed in the liver. D) mRNA expression of the inflammatory mediators *Il1b*, *Tnf*, and *Cxcl2* in hepatic tissue. *Cxcl2* increase leads to the infiltration of granulocytes, as shown in the figure. E) mRNA expression of the inflammatory mediators *Il1b*, *Il6*, and *Tnf* in splenic tissue, characterized by the presence of megakaryocytes.

As previously demonstrated for the non‐targeted stealth liposomes based on PEG‐(DSPE)_2_,^[^ ^17^, ^18^ ^]^ also the same liposome format carrying the Fab’_TRZ_ as targeting moiety showed an improved pharmacokinetic profile with respect to SL‐DXR and SIL‐DXR (**Figure** **4**A and **Table** **4**). All three formulations showed pharmacokinetic advantages when compared with DXR, as expected. SSIL2 could improve DXR pharmacokinetics more than SL and SIL, since elimination half‐life (t1/2 beta) and AUC were significantly higher than in rats treated with untargeted liposomes and SIL, whereas CL was significantly lower, indicating the better drug disposition obtained with the administration of SSIL2.

**Table 4:** Pharmacokinetic parameters (PK Solver 2.0 software).

Parameter	DXR	SL‐DXR	SIL‐DXR	SSIL2‐DXR
*t*_1/2 alpha_ [h]	0.573 ± 0.360	0.260 ± 0.077	0.933 ± 0.348^**^	1.263 ± 0.336^***^
*t*_1/2 beta_ [h]	1.318 ± 0.812^†^	12.23 ± 2.22	19.03 ± 4.08	34.54 ± 8.07^****^, ^###^
CL [mL (h kg)^−1^]	48 446 ± 17 948^†^	35.16 ± 1.40	22.34 ± 1.85^****^	15.11 ± 2.82^****^, ^###^
*V*_ss_ [mL kg^−1^]	56 584 ± 7722^†^	685.24 ± 137.85	550.20 ± 79.97	598.46 ± 94.59
AUC_0‐inf_ [µg (mL h)^−1^]	0.060 ± 0.031^†^	69.07 ± 0.90	112.50 ± 8.42^**^	169.78 ± 2331^****^, ^###^

** *p* < 0.01;

*** *p* < 0.001;

**** *p* < 0.0001 versus SL‐DXR;

^###^ *p* < 0.001 versus SIL‐DXR; one‐way ANOVA followed by Tukey post hoc test;

^†^ The parameters calculated for the three formulations were all significantly different than those calculated for DXR (*p* < 0.0001); one‐way ANOVA followed by the Dunnett post hoc test.

In order to evaluate the safety profile of the liposomal formulations, we evaluated full blood count and plasma biochemistry and performed the histological analysis of the main organs of the animals involved in this study. The results of the hematological and biochemical analyses, performed at the moment of sacrifice (Tables S1 and S2, Supporting Information) suggest that DXR‐treated animals were anemic (red blood cells, hemoglobin, and hematocrit were decreased versus controls, *p* < 0.01), while the same effect was not observed in rats treated with the DXR‐loaded liposomal formulations. Anemia is one of the common side effects of conventional chemotherapy,^[^ ^34^ ^]^ and these results confirmed that the drug encapsulation within the immunoliposomes could effectively prevent or at least reduce this side effect.

In addition, a reduction of plasma levels in SL and SIL‐treated rats (*p* < 0.05 versus control) was observed, which might suggest the risk of hepatotoxicity (Table S2, Supporting Information). Liver damage caused by these two formulations was confirmed by histological analysis, showing multiple granulomatous lesions, sometimes associated with apoptotic bodies, in the liver of rats administered with SL‐ and SIL‐DXR (Figure 4B). Liver histology indicated that these lesions were characterized by the presence of neutrophils, particularly evident in rats treated with SIL‐DXR. Interestingly, in rats treated with SSIL2‐DXR only a small and isolated granuloma could be observed in the otherwise healthy livers (Figure 4B).

Accordingly, the mRNA hepatic expression of Cxcl2, a chemokine involved in the recruitment of neutrophils,^[^ ^35^ ^]^ was significantly higher in SL‐ and SIL‐treated animals with respect to control and SSIL2‐DXR (Figure 4D). Liver inflammation was confirmed by the increase of the mRNA expression of two generic pro‐inflammatory cytokines, that is, Il1b and Tnf, which increased significantly only in the livers of SIL‐treated rats (*p* < 0.001), and was comparable to controls in SSIL2‐treated rats (Figure 4D).

Animals treated with SL‐DXR and SIL‐DXR showed histological alterations also in the spleen (Figure 4C), that is, variable degree of histiocytosis associated with a marked increase in extramedullary hematopoiesis (megakaryocytosis, especially in SIL‐treated rats, Figure 4E). Interestingly, in rats treated with SSIL2‐DXR only a slight increase of hematopoiesis could be observed in the splenic tissue. These results were in accordance with the mRNA splenic expression of three pro‐inflammatory cytokines, that is, *Il1b*, *Il6*, and *Tnf*, which all increased significantly in SIL‐treated rats (Figure 4E). The other organs (i.e., heart, lungs, brain) did not show any pathological alteration in all treatment‐groups.

### 3.5 Activity Evaluation in a Zebrafish Xenograft Model of Ovarian Cancer

**Figure 5:**
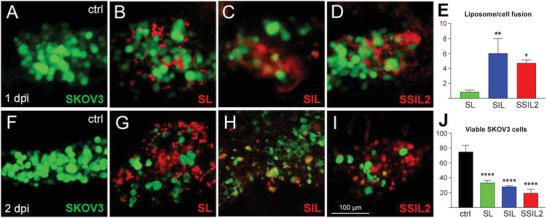
Evaluation of liposome formulations in SKOV3‐engrafted zebrafish embryos. A–E) By 1 dpi, liposomes (red) have reached SKOV3 cells (green; untreated control in (A)), with different degrees of fusion ((B)–(D), chart in (E)). F–J) At 2 dpi, the tumor mass is reduced with G–I) all formulations, compared to the untreated control ((F); chart in (J)). All images are lateral views of the embryonic yolk, anterior to the left. *N* = 7 measures/condition; ^*^ *p* < 0.05, ^**^ *p* < 0.01, ^****^ *p* < 0.0001.

The zebrafish is a small teleost belonging to the Cyprinidae family. Considering the large number of eggs produced after breeding, the fast embryonic development, and the gene and anatomic conservation with mammals, the zebrafish has emerged as an excellent platform to perform cancer xenografts and drug screenings.^[^ ^36^, ^37^, ^38^ ^]^ In light of this, tropism of different liposome formulations toward cancer cells was evaluated in zebrafish embryos xenotransplanted with SKOV3 cells and, in a separate yolk area, microinjected with SL, SIL, or SSIL2. Control conditions were represented by SKOV3 or SSIL2 injected alone. To evaluate the different efficiencies of our liposome formulations, we decided to inject them in a zebrafish yolk sack at 2 dpf together with SKOV3 cells. A preliminary evaluation on liposome‐induced cell toxicity was performed by comparing the survival of injected embryos and uninjected controls. These tests, performed in triplicate, showed the following survival ranges after 1‐day post‐injection (dpi): 100% for uninjected embryos, 93–100% for SKOV3‐injected embryos, 64–77% for SKOV3+SL, 51–52% for SKOV3+SIL, 52–58% for SKOV3+ SSIL2, and 76–78% for SSIL2 alone, suggesting that all liposome formulations can exert some toxic effects during zebrafish embryonic development. Taking into consideration this embryonic sensitivity, we limited our observation window to 2 dpi. Tracking liposomes and tumor cells positions within this time frame, we could observe that all formulations could reach SKOV3 cells by 1 dpi, with more efficient fusion for SIL and SSIL2, compared to SL (**Figure** **5**A–E), inducing a 56% (for SL), 63% (SIL), and 75% (SSIL2) reduction of the tumor mass by 2 dpi (Figure 5F–J). Although all formulations were likely used at a concentration range fairly toxic for zebrafish embryos, they nonetheless demonstrated in vivo their ability to rapidly colocalize with the target cells, inducing a significant decrease of the original tumor size (with SSIL2 ≥ SIL > SL efficiency), compared to liposome‐untreated controls.

### 3.6 Efficacy of Targeted and Untargeted Liposomes in an Ovarian Cancer Xenograft Model

In order to assess the antitumor efficacy of DXR‐loaded SL, SIL, and SSIL2, a mouse model of HER2+ ovarian cancer was set up, by xenografting SCID immunodeficient mice with HER2+ SKOV3 cells. Such xenograft model has been also selected because a study investigating seventeen ovarian cancers suggested that the features of SKOV‐3 tumors (growth, invasiveness, capability of creating ascites) are virtually identical when cells are implanted either in the subcutaneous tissue or intraperitoneally,^[^ ^39^ ^]^ thus suggesting that similar results could be obtained when studying immunoliposomes in this setting.

**Figure 6:**
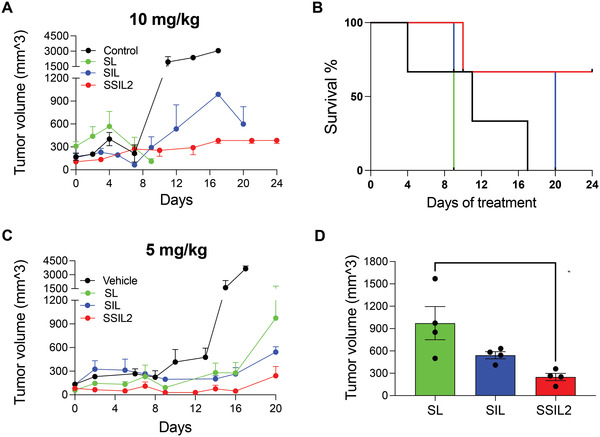
In vivo anticancer activity in SKOV3 ovarian cancer xenograft mouse model. A) Tumor growth and B) Kaplan–Meier curve of xenograft mice treated with 10 mg kg^−1^ (DXR equiv.). C) Tumor growth and D) tumor volumes at sacrifice of xenograft mice treated with the formulations at the lower DXR dose (5 mg kg^−1^ equiv.).

The liposomes have been administered when the tumor mass became palpable in the flank. The treatments have been administered every 7 days with three doses of either 10 or 5 mg kg^−1^ (DXR equivalents). **Figure** **6** clearly shows that, although effective in containing cancer growth (Figure 6A), the 10 mg kg^−1^ dose led to toxicity in mice treated with SL and SIL, so the humane endpoints were reached earlier than the experimental endpoint for both formulations (Figure 6B). This systemic toxicity was significantly more severe for untargeted SL since the median survival of mice treated with this formulation was even lower than that of untreated control mice. On the other hand, besides being well tolerated, thereby confirming the toxicological observations obtained in rats, SSIL2 displayed a marked anticancer activity, allowing most of the treated mice (66%) to reach the experimental endpoint, which was fixed to 24 days of survival, maintaining the tumor volume inferior to 1000 mm^3^. The lower dose (5 mg kg^−1^) (Figure 6C) was well tolerated by all mice since they all reached the experimental endpoint, which was fixed at 20 days. The two targeted formulations were more effective than SL, since the tumor volume was lower at the moment of sacrifice in SIL‐ and SSIL2‐treated mice, and this difference reached statistical significance versus untargeted liposomes for SSIL2 (Figure 6D).

### 3.7 Effect of Liposomes and Immunoliposomes on the TAMs

**Figure 7:**
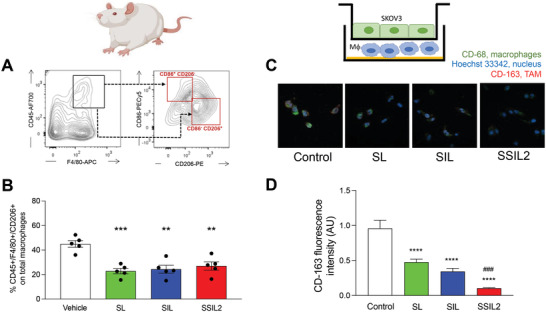
Study of liposomes effect on TAMs. Gating strategy used for the A) identification and B) quantification of TAMs in the in vivo study. C,D) In vitro effect of the treatments on CD68+/CD163+ MΦ cells obtained by coculturing THP‐1 with SKOV3 cells. Magnification: 63×. ^*^ *p* < 0.05, ^**^ *p* < 0.01, ^***^ *p* < 0.001, ^****^ *p* < 0.0001 versus vehicle treated mice or control cells, ^####^ *p* < 0.0001 versus control cells.

The effect of the liposomal treatments on the amount of TAMs (CD45+/F4/80+/CD206+cells)^[^ ^40^ ^]^ inside the tumor masses was evaluated by FACS analysis (**Figure** **7**). The mice were sacrificed 20 days after the treatment with the liposomal formulations (5 mg kg^−1^ of DXR equiv.) and the tumors were collected. Notably, all the treatments were able to reduce the number of CD45+/F4/80+,CD206+ cells present in the tumor mass (Figure 7B). We confirmed this effect also in vitro, in a coculture of SKOV3 cells and THP1‐derived MΦ macrophages, where we observed that SL, SIL, and SSIL2 could reduce the expression of the TAM marker CD163, and that this effect was particularly evident when cells were treated with SSIL2 (Figure 7C,D). However, although the phenotype of protumoral macrophages infiltrating the tumor seems to be affected by the treatments, neither a direct correlation with efficacy nor a peculiar effect of the targeting agent could be observed in vivo. However, considering the multifaceted role of TAMs in cancer^[^ ^41^ ^]^ and their still debated relationship with therapy outcomes, these results pose new questions about the role of untargeted and targeted nanomedicines in modulating the phenotype of the immune cells infiltrating the tumors. Further studies are needed to elucidate the role of liposomes and immunoliposomes in the modulation and immunogenicity of the immune tumor microenvironment.

## 4 Conclusions

A new liposomal formulation endowed with enhanced stability in biological fluids and persistency of the targeting molecules on the surface of liposomes was studied. The Fab’ fragment of TRZ was selected to target with high‐affinity the HER2‐overexpressing cancer cells, a condition characterizing about 20–30% of invasive breast and ovarian carcinomas.^[^ ^16^ ^]^ We named this formulation super stealth immunoliposomes (SSIL2). The longer half‐life and reduced clearance rate of SSIL2, when compared to the classic SL approach, proved the stronger stabilizing effect of PEG‐bi‐lipids derivatives over PEG‐mono‐phospholipid. In vitro cell‐culture studies performed on HER2+ cell lines (BT474, SKOV3, and SKBR3) showed that DXR‐loaded SSIL2 were effectively internalized in the cells and, consequently, more effective in reducing cell viability than the non‐targeted formulation (SL). The SSIL2 performances in reaching and reducing cancer cells were also confirmed in vivo, in a zebrafish xenotransplantation setup. Additionally, SSIL2 evidenced the highest intracellular uptake (even in the cell nucleus), thus proving the beneficial contribution of the TRZ's Fab’ fragment, stably anchored on the surface of liposomes through PEG‐bi‐lipids. Interestingly, the liposomal stabilization offered by the branching structure, which binds two phospholipid units per polymer chain, improved also the toxicological profile of the liposomes. The non‐targeted SL and targeted SIL induced significant histological and molecular alterations in the liver, a tissue rich in reticuloendothelial cells remarkably able to promote hepatic deposition of such liposomes. Conversely, SSIL2 caused extremely limited histological alterations in the liver, as also confirmed by biochemical analyses and mRNA expression of inflammatory mediators.

Overall, we demonstrated that the key strategy of stabilizing the PEG layer on the surface of liposome vesicles through the doubling of the phospholipid units per polymer chain allowed us to extend the advantages of PEG shielding, which have already brought into the market the stealth liposomes, such as Doxil. Furthermore, even the targeting approach could benefit from such steady PEG/phospholipid bilayer interaction achieving better in vitro and in vivo outcomes with respect to a similar liposomal formulation in which the targeting Fab’ was anchored through a single phospholipid unit (SIL). We might speculate that during the biodistribution a percentage of the PEG‐DSPE and Fab’_TRZ_‐PEG‐DSPE linked on the classic immunoliposomes (SIL) are detached over time, thus yielding both a general destabilization of the vesicles, which become more prone to interact with plasma proteins and a reduced number of Fab’ on the liposome surface, thereby reducing the targeting properties of the liposomes. Both these issues are ascribed to the same cause: a weak interaction of the PEG chains with the phospholipid bilayer, which is mediated by a single phospholipid per polymer. The doubling of the number of phospholipids per PEG chain, as here studied for SSIL2, offered an increased hydrophobic anchor that promoted a stable interaction of the PEG chains and the targeting molecules.

In conclusion, in light of the results of this study, super stealth immunoliposomes represent a smart strategy to improve the efficacy and tolerability of conventional cancer chemotherapy.

## Conflict of Interest

The authors declare no conflict of interest.

## Supporting information

Supporting Information

Supplemental Movie 1

Supplemental Movie 2

Supplemental Movie 3

## Data Availability

The data that support the findings of this study are available from the corresponding author upon reasonable request.
