# Supplementary material for: Anti‐HER2 Super Stealth Immunoliposomes for Targeted‐Chemotherapy
Source: Adv Healthc Mater. 2023 Aug 30;12(29):2301650. doi: 10.1002/adhm.202301650 (PMC11469322; doi:10.1002/adhm.202301650)
Supplement: Supplementary file 1 — Supporting Information [file ADHM-12-2301650-s004.pdf]

ADVANCED HEALTHCARE MATERIALS

Supporting Information

for *Adv. Healthcare Mater.*, DOI 10.1002/adhm.202301650

Anti-HER2 Super Stealth Immunoliposomes for Targeted-Chemotherapy

Elena Canato, Antonella Grigoletto, Ilaria Zanotto, Tommaso Tedeschini, Benedetta Campara, Giovanna Quaglio, Giuseppe Toffoli, Delia Mandracchia, Alberto Dinarello, Natascia Tiso, Francesco Argenton, Katia Sayaf, Maria Guido, Daniela Gabbia, Sara De Martin and Gianfranco Pasut**

Supporting Information

Anti-HER2 super stealth immunoliposomes for targeted-chemotherapy

Elena Canato1‡, Antonella Grigoletto1‡, Ilaria Zanotto1, Tommaso Tedeschini1, Benedetta Campara1, Giovanna Quaglio1, Giuseppe Toffoli2, Delia Mandracchia3, Alberto Dinarello4, Natascia Tiso4, Francesco Argenton4, Katia Sayaf5, Maria Guido6,7, Daniela Gabbia1, Sara De Martin1, Gianfranco Pasut1**

PEG$_{5kDa}$-(DSPE)$_2$ ^{1}H-NMR spectra (400 MHz, CDCl$_3$) δ (ppm): 0.88 (t, 11.60 H, -CH_3; DSPE); 1.25 (broad s, 109.37 H, -CH_2; DSPE); 1.58 (m, 7.69 H, -CH_2-CH$_2$-COO-; DSPE); 2.28 (t, 7.11 H, -CH_2-COO-; DSPE); 3.38 (s, 3 H, -OCH_3; PEG); 3.5-3.7 (broad s, 460 H, -O-CH_2-CH_2-; PEG) and 5.22 (m, 1.47 H, -CH-; glycerol, DSPE) (Fig. S1A). DOSY analysis confirmed that DSPE molecules were covalently attached to the polymer backbone since all the protons in the spectrum have the same diffusion coefficient (D= $3{\cdot}10^{-6}$ cm^2/s) (Fig. S1B). The average yield of DSPE coupling was 96.67%, based on the ratio between the experimental value of -CH_3 DSPE's protons (11.60) with respect to the theoretical value (12.00). PEG$_{5kDa}$-(DSPE)$_2$ chemical structure is reported in Fig. S1C.

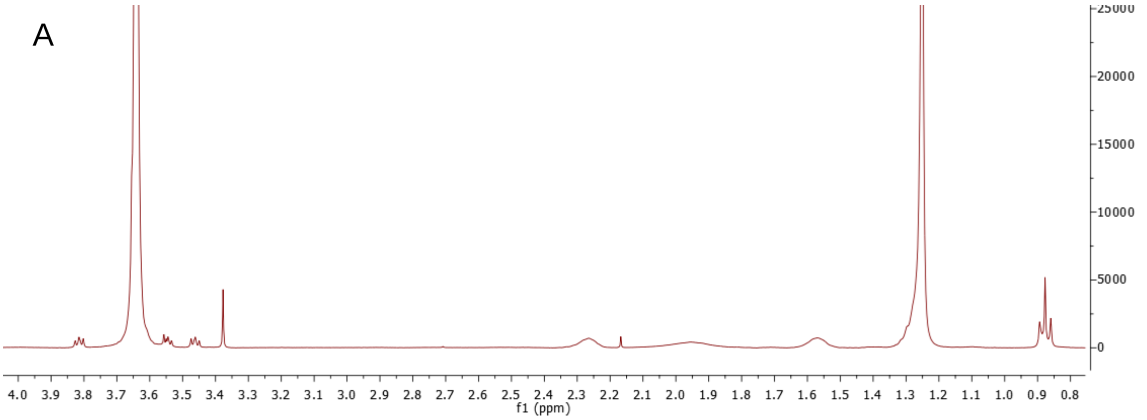

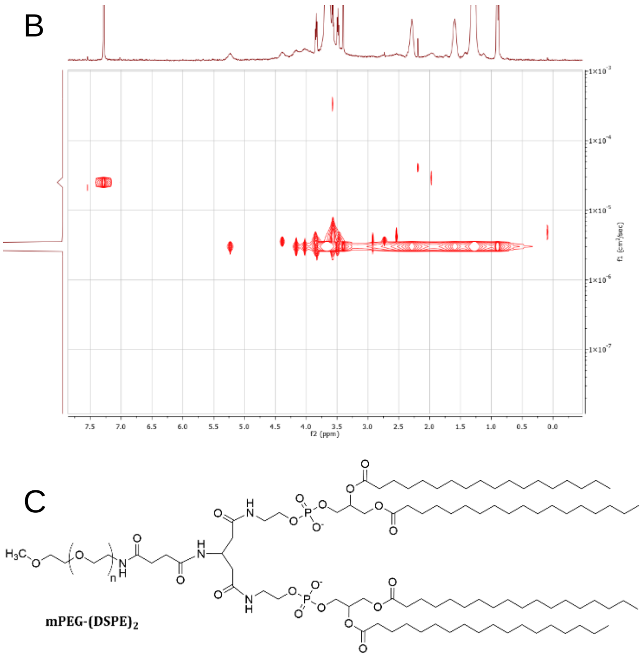

Figure S1. ^{1}H-NMR (A) and DOSY ^{1}H-NMR (B) spectra and (C) chemical structure of PEG_{5kDa}-$(DSPE)_2$.

Boc-PEG-$(DSPE)_2$, ^{1}H-NMR spectra (400 MHz, $CDCl_3$) δ (ppm): 0.88 (t, 11.50 H, -CH_3; DSPE); 1.25 (broad s, 104.47 H, -CH_2; DSPE); 1.44 (s, 9 H, -COO-$(CH_3)_3$; Boc); 1.58 (m, 7.11 H, -CH_2-CH_2-COO-; DSPE); 2.28 (t, 6.95 H, -CH_2-COO-; DSPE); 3.5-3.7 (broad s, 520 H, -O-CH_2-CH_2-; PEG) and 5.22 (m, 1.61 H, -C*H*-; glycerol, DSPE). The average yield of DSPE coupling was 95.83%, based on the ratio between the experimental value of -CH_3 DSPE's protons (11.50) with respect to the theoretical value (12.00). The removal of Boc protecting group in acidic conditions was confirmed by the disappearance of the corresponding peak at 1.44 ppm as indicated by the arrows in Fig. S2A. Boc-PEG-$(DSPE)_2$ chemical structure is reported in Fig. S2B.

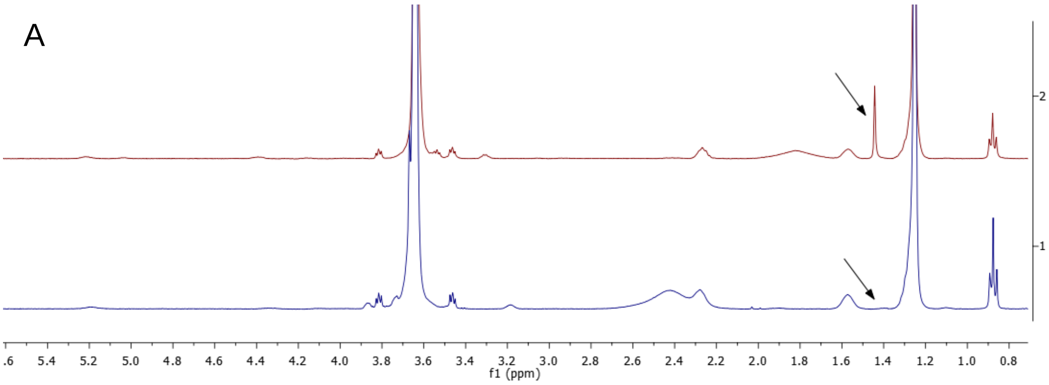

B

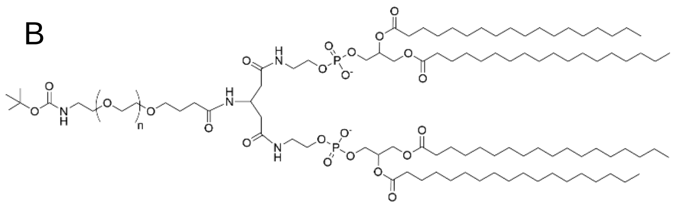

Figure S2. (A) ^{1}H-NMR spectra of Boc-PEG-(DSPE)$_2$ (in red) and H_2N-PEG-(DSPE)$_2$ (in blue); (B) chemical structure of Boc-PEG-(DSPE)$_2$.

The maleimide group was introduced by conjugation with the heterobifunctional cross-linker BMPS to allow the following reaction with the free sulfhydryl groups of the Fab' fragment, through the formation of a covalent thioether bond. MAL-PEG-(DSPE)$_2$ ^{1}H-NMR spectra (400 MHz, $CDCl_3$) δ (ppm): 0.88 (t, 11.36 H, -C*H*$_3$; DSPE); 1.26 (broad s, 101.20 H, -C*H*$_2$; DSPE); 3.5-3.7 (broad s, 520 H, -O-C*H*$_2$-C*H*$_2$-; PEG) and 6.69 (s, 1.46 H, -C*H*=C*H*-; MAL), consequently the BMPS coupling efficiency was 73.0% (Fig. S3A). DOSY analysis confirmed a single diffusion coefficient (D= $8.35\cdot10^{-7}$ cm^2/s) meaning that both DSPE molecules and maleimide group were covalently attached to the polymer backbone (Fig. S3B). The chemical structures of BMPS and MAL-PEG-(DSPE)$_2$ are reported in Fig. S2C.

A

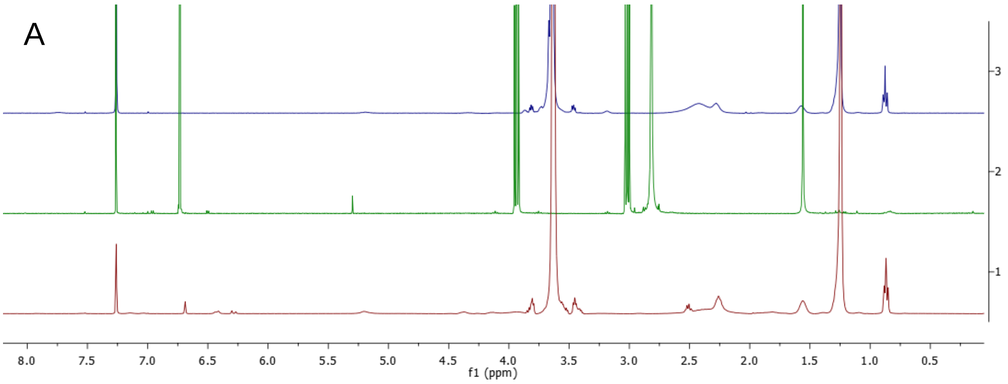

B

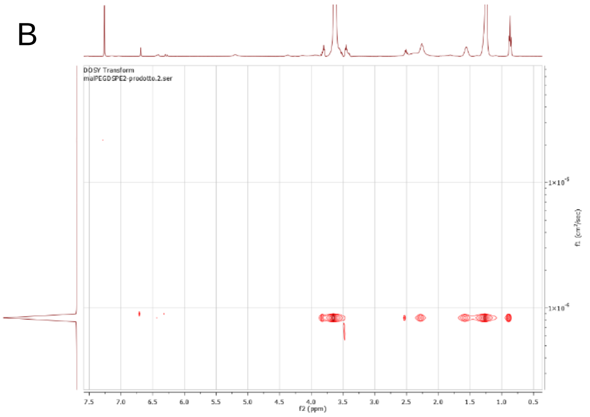

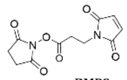

C

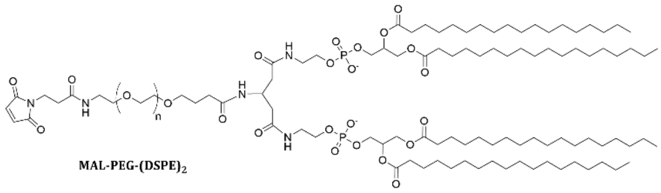

Figure S3. (A) ^{1}H-NMR spectra of H_2N-PEG-$(DSPE)_2$ (in blue), BMPS (in green) and MAL-PEG-$(DSPE)_2$ (in red). BMPS showed the following δ (ppm) peak values: 1.56 (s, residual water), 2.82 (s, 4H, -CH_2; NHS), 3.02 (t, 2H, -CH_2-COO-), 3.94 (t, 2H, -CH_2-CH_2-COO-) and 6.73 (s, 2H, -CH=CH-; MAL). (B) DOSY ^{1}H-NMR spectra and (C) chemical structures of BMPS and MAL-PEG-$(DSPE)_2$.

F(ab')$_2$ and Fab' were purified by size exclusion chromatography, showing retention volumes of 13 ml and 14.5 ml, respectively (Fig. S4). The collected proteins were analyzed by SDS-PAGE (Fig. S4) and appeared as single bands at about 100 kDa and 50 kDa, respectively, whereas Trastuzumab evidenced a molecular weight of about 150 kDa, as expected. These results were confirmed by MALDI-TOF analysis (Fig. S5): 148720.7 Da, 97628.0 Da, and 48772.2 Da, respectively for Trastuzumab, F(ab')$_2$, and Fab'.

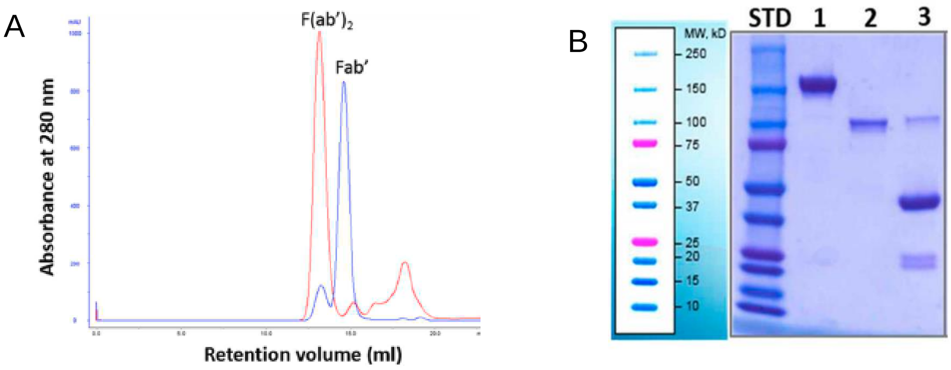

Figure S4. (A) Analytical chromatographic profiles of Fab' and F(ab')$_2$ reaction mixtures by gel filtration chromatography on a Superose 12 10/300 GL column (λ=280 nm). (B) Characterization by SDS-PAGE (4-15%) in non-reducing conditions of protein standards (STD), (1) Trastuzumab, (2) F(ab')$_2$ and (3) Fab'.

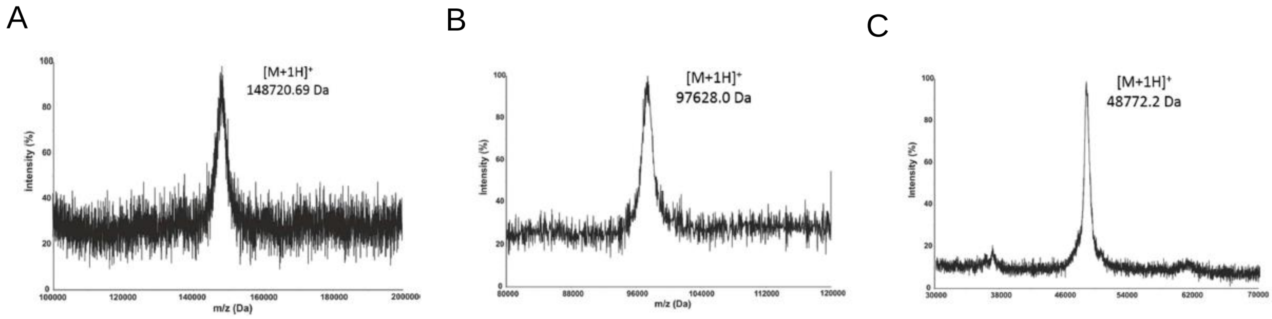

Figure S5. Determination of (A) Trastuzumab, (B) F(ab')$_2$, and (C) Fab' molecular weights by MALDI-TOF.

The reaction mixtures of Fab' PEGylation with either MAL-PEG$_{5kDa}$-DSPE or MAL-PEG$_{5kDa}$-(DSPE)$_2$ were evaluated by SDS-PAGE (Fig. S6). PEG confers a higher apparent MW to PEGylated proteins in SDS-PAGE, owing to its large hydrodynamic volume. As demonstrated in Fig. S6 both MAL-PEG$_{5kDa}$-DSPE (lane 4') and MAL-PEG$_{5kDa}$-(DSPE)$_2$ (lane 6') run as the protein marker of 10 kDa. Consequently, the PEGylated Fab' fragments (Fab'-PEG$_{5kDa}$-(DSPE) in lane 5-5' and Fab'-PEG$_{5kDa}$-(DSPE)$_2$ in lane 7-7' appeared as a main band at an apparent MW of 75 kDa, corresponding to the biconjugate, and a minor band at about 60 kDa corresponding to the monoconjugate. Both reduced thiol groups of Fab' fragment, coming from the hinge region of the heavy chain of TRZ, are exposed and available for PEGylation, therefore the biconjugate is readily obtained. Excessive Fab' reduction in the preparation step can yield the reduction of the interchain disulphide bond, leading to the appearance of two minor bands at 20 and 25 kDa, corresponding to the light and heavy chain respectively.

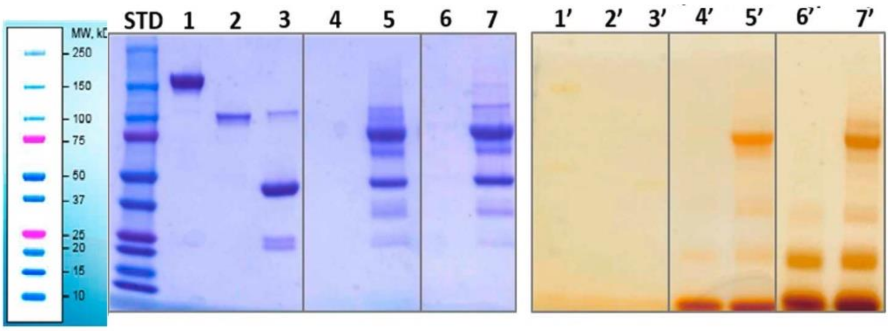

Figure S6. SDS-PAGE (4-15%) characterization in non-reducing conditions of (STD) protein standards, (1-1') Trastuzumab, (2-2') F(ab')$_2$, (3-3') Fab', (4-4') commercial MAL-PEG$_{5kDa}$-DSPE, (5-5') reaction mixture of Fab' PEGylated with MAL-PEG$_{5kDa}$-DSPE, (6-6') MAL-PEG$_{5kDa}$-(DSPE)$_2$, (7-7') reaction mixture of Fab' PEGylated with MAL-PEG$_{5kDa}$-(DSPE)$_2$. The gels were first stained with iodine for PEG visualization (right), then bleached with sodium ascorbate, and then permanently stained with Coomassie blue to visualize the proteins (left).

Purified immunoliposomes were assessed by SDS-PAGE analysis, according to Coomassie blue and silver staining for protein revelation (Fig. S7).

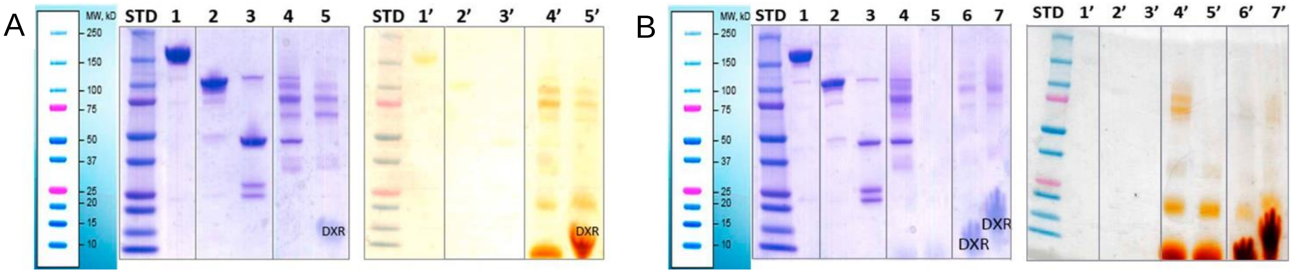

Figure S7. SDS-PAGE (4-15%) characterization in non-reducing conditions of (A): (STD) protein standards, (1-1') Trastuzumab, (2-2') F(ab')$_2$, (3-3') reaction mixture of F(ab')$_2$ reduction with cysteamine, (4-4') Fab' PEGylation with MAL-PEG$_{5kDa}$-DSPE (reaction mixture), (5-5') SIL after purification by chromatography on CL-4B column; (B): (1-1') Trastuzumab, (2-2') F(ab')$_2$, (3-3') reaction mixture of F(ab')$_2$ reduction with cysteamine 10 mM, (4-4') Fab' PEGylation with MAL-PEG$_{5kDa}$-(DSPE)$_2$ (reaction mixture), (5-5') MAL-PEG$_{5kDa}$- DSPE$_2$; (6-6')-(7-7') 2,5 and 5µl respectively of SSIL$_2$ after purification by gel filtration chromatography on CL-4B column. The gels were first stained with iodine for PEG visualization (right), then bleached with sodium ascorbate, and then permanently stained with Coomassie blue to visualize the proteins (left).

The stability of DXR-loaded liposomes was monitored for 2 months by incubating the formulations at 4 and 25 °C.

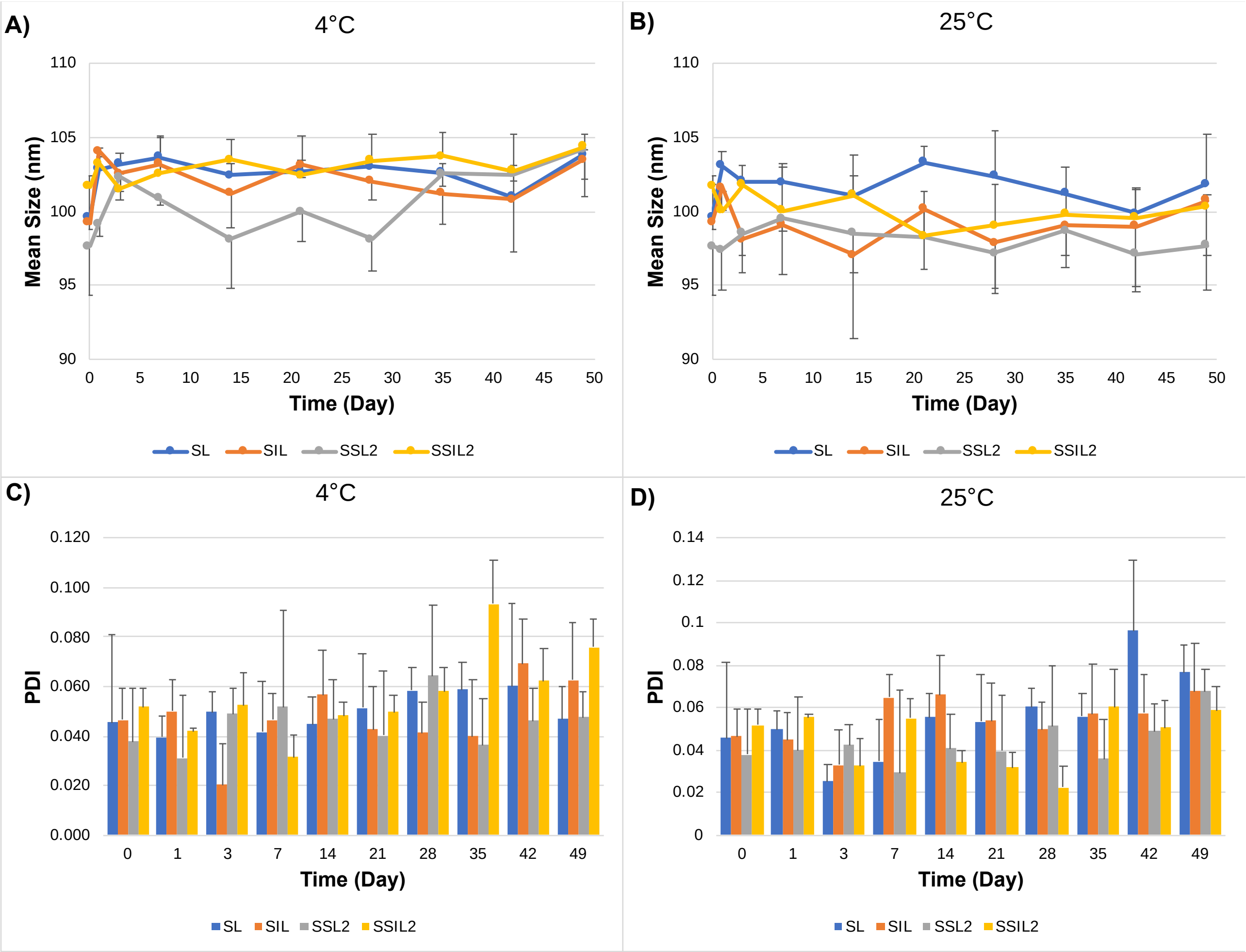

Figure S8. Long-term stability of doxorubicin-loaded SL, SIL, SSL2, and SSIL2, expressed in terms of vesicles mean size (nm) at 4°C (A) and 25 °C (B) and PDI at 4°C (C) and 25 °C (D).

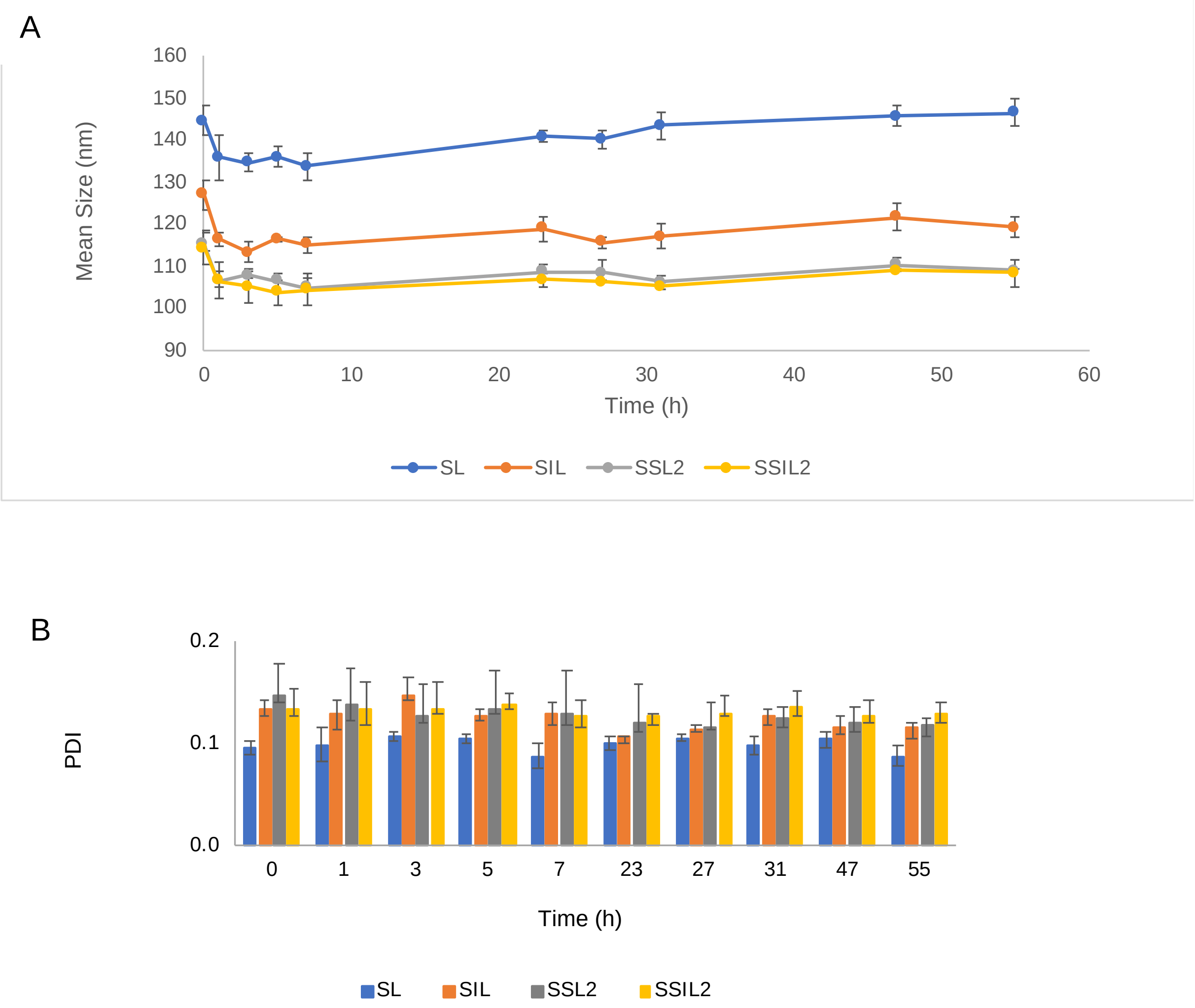

Figure S9. Long-term stability of DXR-loaded SL, SIL, SSL2, and SSIL2, expressed in terms of vesicles mean size (nm) at 37°C (A) and PDI (B).

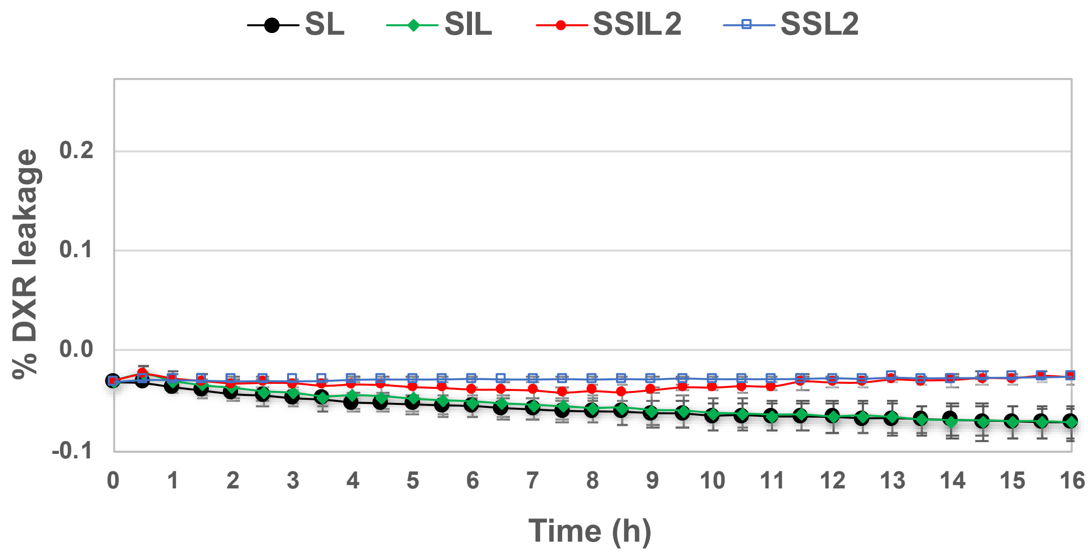

Figure S10. In vitro release profiles of DXR from the liposomal formulations incubated in buffer at pH 7.4 (37°C).

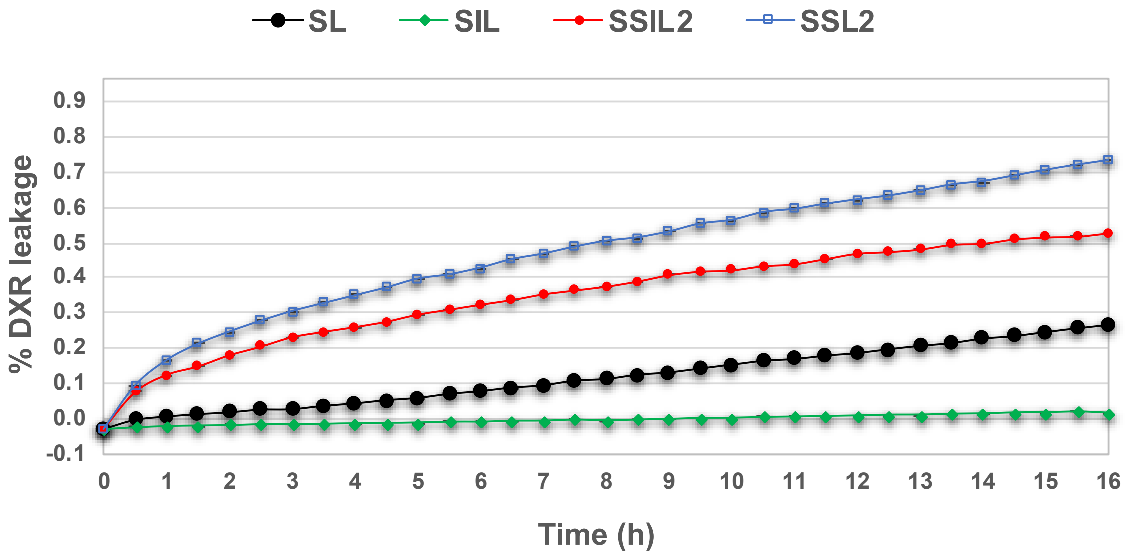

Figure S11. In vitro release profiles of DXR from the liposomal formulations incubated in buffer at pH 5.5 (37°C).

The results of blood and plasma analyses of animals treated with the DXR-loaded liposomal formulations and DXR are shown in tables S1 and S2, respectively.

Table S1. Complete Blood count.

		Controls	***DXR***	***SL***	***SIL***	***SSIL2***
Leukocytes (WBCs)	$x10^9/L$	3.70 ± 1.56	2.05 ± 1.15	2.35 ± 0.35	3.51 ± 1.67	2.56 ± 0.66
Erythrocytes (RBCs)	$x10^{12}/L$	5.45 ± 0.27	4.61 ± 0.53**	5.21 ± 0.06	5.08 ± 0.19	4.93 ± 0.17
Hemoglobin	g/L	112 ± 7	93 ± 10**	105 ± 2	106 ± 4	98 ± 3
Hematocrit	L/L	0.354 ± 0.022	0.286 ± 0.041**	0.331 ± 0.006	0.320 ± 0.007	0.316 ± 0.005
MCV	fL	64.4 ± 3.3	61.8 ± 2.9	63.5 ± 1.0	63.0 ± 1.4	64.1 ± 1.4
MCH	pg	20.5 ± 0.5	20.3 ± 0.4	20.1 ± 0.4	20.8 ± 0.4	19.9 ± 0.4
MCHC	g/L	320 ± 14	329 ± 20	317 ± 1	331 ± 6	310 ± 4
RDW	%	12.6 ± 1.0	11.8 ± 0.5	11.7 ± 0.4	11.7 ± 0.1	11.9 ± 0.2
Platelets (PLTs)	$x10^9/L$	792 ± 295	811 ± 402	439 ± 192	780 ± 91	600 ± 54
**WBC differential count**						
Neutrophils	$x10^9/L$	0.19 ± 0.18	0.21 ± 0.31	0.36 ± 0.24	0.02 ± 0.01	0.22 ± 0.15
%		6.4 ± 6.3	10.1 ± 14.0	16.7 ± 13.5	0.5 ± 0.4	8.3 ± 4.6
Lymphocytes	$x10^9/L$	2.65 ± 1.05	1.68 ± 1.09	1.82 ± 0.62	3.26 ± 1.63	2.25 ± 0.59
%		73.1 ± 5.8	80.8 ± 13.7	76.0 ± 16.6	92.1 ± 2.7	87.8 ± 3.0
Monocytes	$x10^9/L$	0.81 ± 0.57	0.15 ± 0.07	0.15 ± 0.09	0.21 ± 0.03	0.06 ± 0.05
%		19.1 ± 9.00	8.23 ± 5.24	6.53 ± 4.75	6.70 ± 2.35	2.83 ± 2.30
Eosinophils	$x10^9/L$	0.03 ± 0.01	0.02 ± 0.01	0.02 ± 0.03	0.01 ± 0.01	0.02 ± 0.01
%		0.9 ± 0.3	0.9 ± 0.6	0.8 ± 1.1	0.2 ± 0.3	0.9 ± 0.1
Basophils	$x10^9/L$	0.02 ± 0.01	0.01 ± 0.01	0.01 ± 0.01	0.01 ± 0.01	0.01 ± 0.01
%		0.5 ± 0.2	0.1 ± 0.2	0.1 ± 0.2	0.1 ± 0.2	0.2 ± 0.2

**$p<0.01$ vs controls

Table S2. Plasma biochemistry.

		Controls	*DXR*	*SL*	*SIL*	*SSIL2*
Glucose	mmol/L	11.5 ± 1.0	10.7 ± 0.8	12.3 ± 0.6	10.7 ± 0.8	11.6 ± 2.1
Urea	mmol/L	5.4 ± 0.8	4.7 ± 0.2	4.5 ± 0.2	4.8 ± 0.4	5.2 ± 0.9
Creatinine	mmol/L	19 ± 4	16 ± 2	17 ± 1	18 ± 3	17 ± 2
Total bilirubin	mmol/L	0.6 ± 0.2	0.4 ± 0.2	0.6 ± 0.3	0.8 ± 0.2	0.7 ± 0.1
Direct bilirubin	mmol/L	0.3 ± 0.2	0.3 ± 0.2	0.2 ± 0.1	0.4 ± 0.1	0.4 ± 0.3
Sodium	mmol/L	141 ± 2	142 ± 1	142 ± 1	143 ± 1	143 ± 2
Potassium	mmol/L	3.8 ± 1.0	3.9 ± 0.4	3.7 ± 0.2	3.9 ± 0.1	3.7 ± 0.3
Chloride	mmol/L	101 ± 1	102 ± 2	103 ± 1	103 ± 2	102 ± 2
Albumin	g/L	44 ± 3	42 ± 3	37 ± 2*	37 ± 2*	42 ± 2
Calcium	mmol/L	2.76 ± 0.09	2.67 ± 0.09	2.67 ± 0.10	2.64 ± 0.03	2.65 ± 0.06
Inorganic Phosphorous	mmol/L	2.50 ± 0.30	2.45 ± 0.16	1.88 ± 0.26	2.27 ± 0.53	2.64 ± 0.02
AST	U/L	91 ± 36	78 ± 16	67 ± 2	71 ± 5	83 ± 7
ALT	U/L	26 ± 4	20 ± 6	13 ± 2	21 ± 5	30 ± 6
ALP	U/L	176 ± 36	143 ± 36	168 ± 32	143 ± 6	152 ± 6
Total cholesterol	mg/dL	65 ± 7	64 ± 9	78 ± 11	64 ± 9	79 ± 11
Triglycerides	mg/dL	122 ± 73	64 ± 37	49 ± 9	60 ± 27	44 ± 37

*p<0.05 vs controls

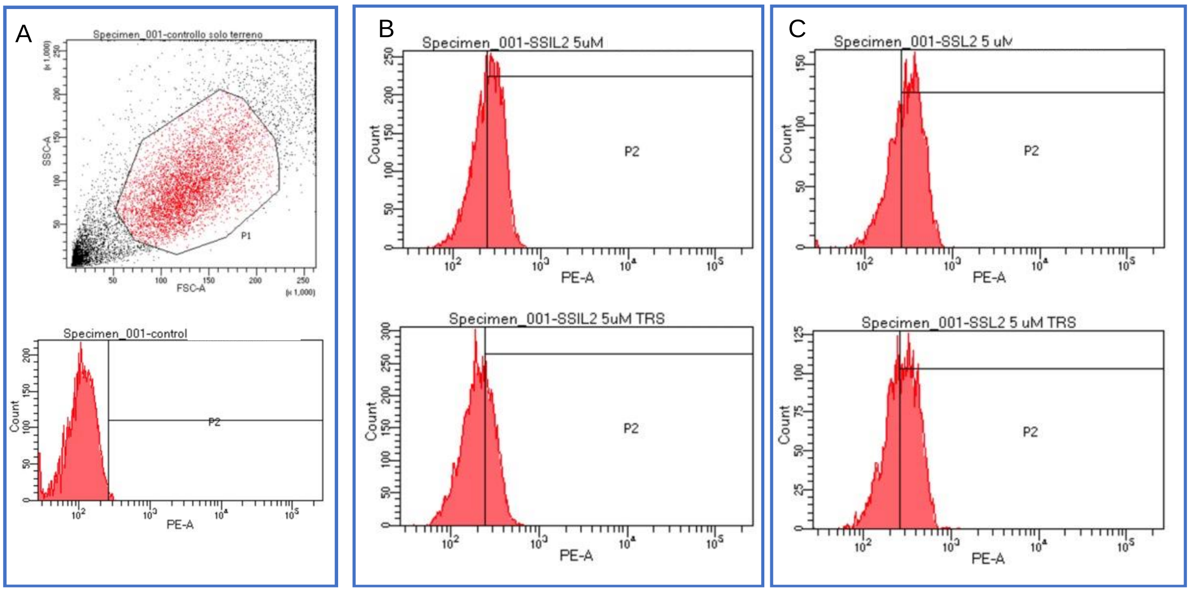

Figure S12. DXR fluorescence shift of SSL2 and SSIL after preincubation with 140 nM trastuzumab (A) Cell selection and distribution of control untreated cells. (B). DXR fluorescence of the SSIL2 (not pretreated with trastuzumab in the upper panel, pretreated with plasma in the lower) and (C) SSL2 (not pretreated with trastuzumab in the upper panel, pretreated with plasma in the lower). To perform this experiment, SKOV-3 cells were seeded

in a 24-well plate (10^5 cell/mL) and after 24h incubated with trastuzumab (140 nM) dissolved in complete medium for 2 h at 37 °C. Then, cells were treated with liposomes (SSL2 or SSIL2) at a concentration of 5 μM in doxorubicin. After 24h, the medium was removed, and cells were trypsinized and centrifuged to obtained single cell suspension. Cells treated with complete medium were used as controls. Liposome uptake in treated cells was assessed by analyzing doxorubicin-positive cells by means of flow cytometry (BD FACSAria III, 10,000 events/sample, ex/em 460/570 nm).

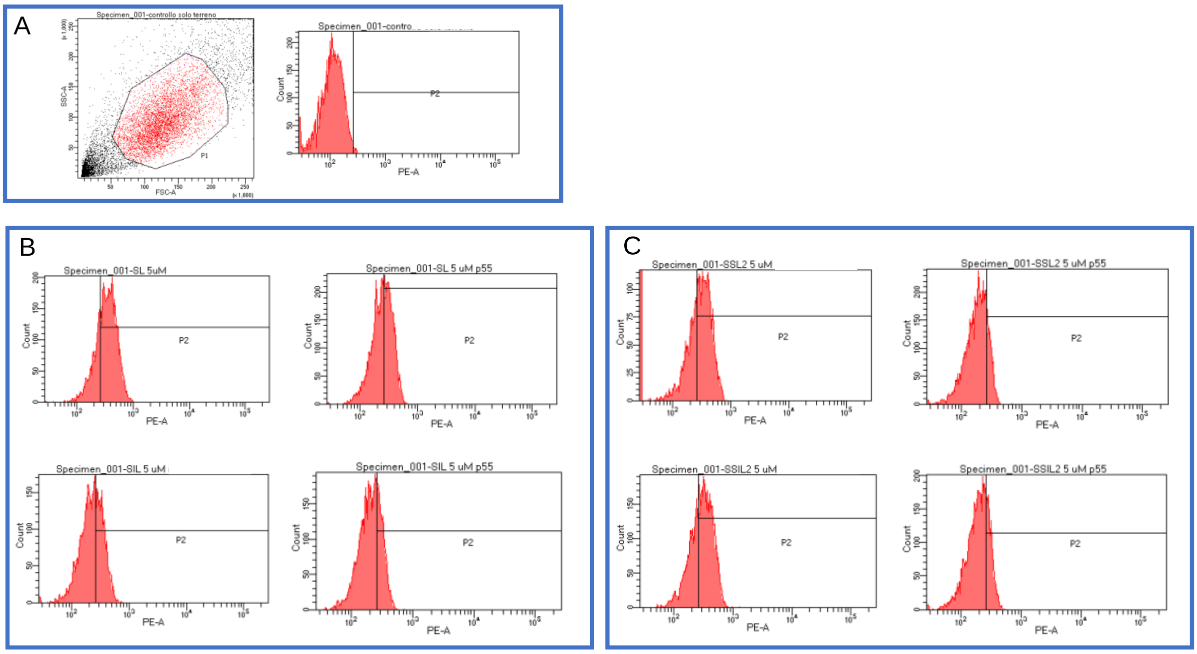

Figure S13. DXR fluorescence shift of SL, SIL, SSL2 and SSIL after preincubation with 55% of rat plasma. (A) Cell selection and distribution of control untreated cells. (B). DXR fluorescence of the stealth formulations SL (upper panel, not pretreated with plasma on the left, pretreated with plasma on the right) and SIL (lower panel, not pretreated with plasma on the left, pretreated with plasma on the right). (C) DXR fluorescence of the SuperStealth formulations SSL2 (upper panel, not pretreated with plasma on the left, pretreated with plasma on the right) and SSIL2 (lower panel, not pretreated with plasma on the left, pretreated with plasma on the right).
